# Type 2 diabetes and pre-diabetes mellitus: a systematic review and meta-analysis of prevalence studies in women of childbearing age in the Middle East and North Africa, 2000–2018

**DOI:** 10.1186/s13643-019-1187-1

**Published:** 2019-11-08

**Authors:** Rami H. Al-Rifai, Maria Majeed, Maryam A. Qambar, Ayesha Ibrahim, Khawla M. AlYammahi, Faisal Aziz

**Affiliations:** 1 Institute of Public Health, College of Medicine and Health Sciences, United Arab Emirates University, P.O. Box 15551, Al Ain, United Arab Emirates; 2 Department of Biology, College of Sciences, United Arab Emirates University, P.O. Box 15551, Al Ain, United Arab Emirates

**Keywords:** Type 2 diabetes, Pre-diabetes mellitus, Women of childbearing age

## Abstract

**Background:**

Investing in women’s health is an inevitable investment in our future. We systematically reviewed the available evidence and summarized the weighted prevalence of type 2 diabetes (T2DM) and pre-diabetes mellitus (pre-DM) in women of childbearing age (15–49 years) in the Middle East and North African (MENA) region.

**Methods:**

We comprehensively searched six electronic databases to retrieve published literature and prevalence studies on T2DM and pre-DM in women of childbearing age in the MENA. Retrieved citations were screened and data were extracted by at least two independent reviewers. Weighted T2DM and pre-DM prevalence was estimated using the random-effects model.

**Results:**

Of the 10,010 screened citations, 48 research reports were eligible. Respectively, 46 and 24 research reports on T2DM and pre-DM prevalence estimates, from 14 and 10 countries, were included. Overall, the weighted T2DM and pre-DM prevalence in 14 and 10 MENA countries, respectively, were 7.5% (95% confidence interval [CI], 6.1–9.0) and 7.6% (95% CI, 5.2–10.4). In women sampled from general populations, T2DM prevalence ranged from 0.0 to 35.2% (pooled, 7.7%; 95% CI, 6.1–9.4%) and pre-DM prevalence ranged from 0.0 to 40.0% (pooled, 7.9%; 95% CI, 5.3–11.0%). T2DM was more common in the Fertile Crescent countries (10.7%, 95% CI, 5.2–17.7%), followed by the Arab Peninsula countries (7.6%, 95% CI, 5.9–9.5%) and North African countries and Iran (6.5%, 95% CI, 4.3–9.1%). Pre-DM prevalence was highest in the Fertile Crescent countries (22.7%, 95% CI, 14.2–32.4%), followed by the Arab Peninsula countries (8.6%, 95% CI, 5.5–12.1%) and North Africa and Iran (3.3%, 95% CI, 1.0–6.7%).

**Conclusions:**

T2DM and pre-DM are common in women of childbearing age in MENA countries. The high DM burden in this vital population group could lead to adverse pregnancy outcomes and acceleration of the intergenerational risk of DM. Our review presented data and highlighted gaps in the evidence of the DM burden in women of childbearing age, to inform policy-makers and researchers.

**Systematic review registration:**

PROSPERO CRD42017069231

## Background

The global burden of type 2 diabetes mellitus (T2DM) is rapidly increasing, affecting individuals of all ages. The global T2DM prevalence nearly doubled in the adult population over the past decade from 4.7% in 1980 to 8.5% in 2014 [1]. The global burden of T2DM in people 20–79 years is further projected to increase to 629 million in 2045 compared to 425 million in 2017 [1]. Low- and middle-income countries will be the most affected with the rise in the burden of T2DM. For the period between 2017 and 2045, the projected increase in the prevalence of T2DM in the Middle East and North Africa (MENA) region is 110% compared to 16% in Europe, 35% in North Africa and the Caribbean, and 62% in South and Central America [1]. Pre-diabetes (pre-DM) or intermediate hyperglycaemia is defined as blood glucose levels above the normal range, but lower than DM thresholds [1]. The burden of pre-DM is increasing worldwide. By 2045, the number of people aged between 20 and 79 years old with pre-DM is projected to increase to 587 million (8.3% of the adult population) compared to 352.1 million people worldwide in 2017 (i.e., 7.3% of the adult population of adults aged 20 to 79 years) [1]. About three quarters (72.3%) of people with pre-DM live in low- and middle-income countries [1].

Pre-DM or T2DM are associated with various unfavorable health outcomes. People with pre-DM are at high risk of developing T2DM [1]. Annually, it is estimated that 5–10% of people with pre-DM will develop T2DM [2, 3]. Pre-DM and T2DM are also associated with early onset of nephropathy and chronic kidney disease [4–7], diabetic retinopathy [6, 8, 9], and increased risk of macrovascular disease [10, 11]. T2DM is also reported to increase the risk of developing active [12] and latent tuberculosis [13]. The rising levels of different modifiable key risk factors, mainly body overweight and obesity, driven by key changes in lifestyle, are the attributes behind the continued burgeoning epidemics of pre-DM and T2DM [14–16]. Women of childbearing age (15–49 years) [17] are also affected by the global rise in pre-DM and T2DM epidemics. Rising blood glucose levels in women of childbearing age has pre-gestational, gestational, and postpartum consequences, including increased intergenerational risk of DM [18].

The total population in 20 countries (Algeria, Bahrain, Djibouti, Egypt, Iran, Iraq, Jordan, Kuwait, Lebanon, Libya, Malta, Morocco, Oman, Palestine, Qatar, Saudi Arabia, Syria, Tunisia, the United Arab Emirates, and Yemen) in the Middle East and North Africa region comprises almost 6.7% (~ 421 million people) of the world’s population, with about 200 million females as of July 1, 2015 [19]. In adults ≥ 18 years, T2DM prevalence rose sharply by 2.3 times in each of the Eastern Mediterranean regions and the African region, between 1980 and 2014 [20]. This sharp increase in these two regions is higher than that reported in the region of the Americas (1.7 times), the European region (1.4 times), and the Western Pacific Region (1.9 times) [20].

Key pre-DM and T2DM risk factors, body overweight and obesity, are highly prevalent in people in the MENA countries. In 2013, the age-standardized prevalence of overweight and obesity among women ≥ 20 years was 65.5% (obese 33.9%) [21]. The high burden of overweight and obesity in several MENA countries attributed to the interrelated economic, dietary, lifestyle behavioral factors. The nutrition transitions and changes in the food consumption habits were supported by the witnessed economic development in most of the MENA countries. For instance, in the past five decades, the economic development in the Arab Gulf countries linked to the discovery of oil and gas reserves led to changes in eating habits towards the consumption of foods rich in fat and calories as well as increasing behavioral habits towards a sedentary lifestyle [22, 23]. This is particularly true with the significant shift from the consumption of traditional low-fat food to fat-rich foods, as well as with a major change from an agricultural lifestyle to an urbanized lifestyle that is often accompanied by decreased levels of physical activity. The urbanized lifestyle increases exposure to fast foods through the high penetration of fast food restaurants serving fat-rich foods, the reliance on automobiles for transport, and the increasing penetration of cell phones, all of which facilitate low levels of physical activity. Globally, physical inactivity is estimated to cause around 27% of diabetes cases [24]. In eight Arab countries, based on national samples, low levels of physical activity in adults ranged from 32.1% of the population in Egypt in 2011–2012 to as high as 67% of the population in Saudi Arabia in 2005 [25]. Furthermore, fruit and vegetable consumption is inversely associated with weight gain [26]. Studies indicated a low intake of fruit and vegetables in some of the MENA countries [27, 28]. The growing burden of the possible risk factors of body overweight and obesity in women may further affect and exacerbate the burden of DM and its associated complications in the MENA countries.

To develop effective prevention and control interventions, there is a need for understanding the actual burden of pre-DM and T2DM epidemics in vital population groups, such as women of childbearing age (15–49 years), in the MENA region. Thus, individual studies need to be compiled and summarized. According to our previously published protocol (with a slight deviation) [29], here, we present the results of the systematically reviewed published quantitative literature (systematic review “1”), to assess the burden (prevalence) of T2DM and pre-DM in women of childbearing age in the MENA region, from 2000 to 2018.

Investing in women’s health paves the way for healthier families and stronger economies. Societies that prioritize women’s health are likely to have better population health overall and to remain more productive for generations to come [30]. Against this background, our review was aimed at characterizing the epidemiology of T2DM and pre-DM in population groups of women of childbearing age in the MENA through (1) systematically reviewing and synthesizing all available published records of T2DM and pre-DM and (2) estimating the mean T2DM and pre-DM prevalence at national, sub-regional, and regional levels, from January 2000 to July 2018. The findings of the review fill an evidence gap to inform policy-makers on the epidemiologic burden of T2DM and pre-DM in women of childbearing age.

## Methods

Following our published protocol [29] that is registered with the International Prospective Registry of Systematic Reviews (PROSPERO registration number “CRD42017069231” dated 12/06/2017), we reported here systematic review “1”. This review adheres to the Preferred Reporting Items for Systematic Review and Meta-Analysis (PRISMA) 2009 guidelines [31–33]. The PRISMA checklist is provided in the Additional file 1.

### Data source and search strategy

To identify eligible studies on T2DM and pre-DM prevalence measures in MENA countries, we implemented a comprehensive computerized search of six electronic databases (MEDLINE, EMBASE, Web of Science, SCOPUS, Cochrane library, and Academic Search Complete) from January 1, 2000, to July 12, 2018, using variant Medical Subject Headings (MeSH) and free-text (Text) terms. The detailed search strategy is presented in an additional box file (see Additional file 2). We also hand-searched the reference lists of eligible studies for further studies that might have been missed.

We defined the participants, exposure, comparator, outcome(s), and type of study “PECO(T)”. The PECO(T) statement provides the framework for the identification and selection of studies for inclusion [34]. As we were looking for prevalence studies, we only considered participants and the outcomes.

### Inclusion and exclusion criteria

*Participants*: Women of childbearing age were defined according to the World Health Organization (WHO) as women aged between 15 and 49 years (thereafter, women of childbearing age) [35]. Pregnant women were also considered in this review as long as they were tested for T2DM and/or pre-DM according to what was reported in the individual studies.

*Outcomes*: T2DM and pre-DM. The included studies should have reported quantitative or calculable pre-DM or T2DM prevalence estimate(s) in women of childbearing age regardless of the sample size, pregnancy status, or pre-DM/T2DM ascertainment methodology, in any of the 20 MENA region countries [36]. We excluded studies of self-reported pre-DM/T2DM not supported with either anti-DM medications or a documented diagnosis. We also excluded studies on metabolic syndrome as long as there was no clear information on the proportion of women of childbearing age with pre-DM or T2DM. Studies were also excluded if they pooled women of childbearing age with pre-DM/T2DM with other non-communicable diseases in the same category, or together with males, or for each gender separately but without age stratification. We excluded studies with incalculable pre-DM/T2DM prevalence after attempting to contact the authors at least twice with no response.

*Types of studies*: We included observational studies if they were cross-sectional, comparative cross-sectional, case-control (not comparing T2DM/pre-DM vs. no T2DM/pre-DM), or cohort study designs. We excluded observational studies of other study designs.

**Fig. 1:**
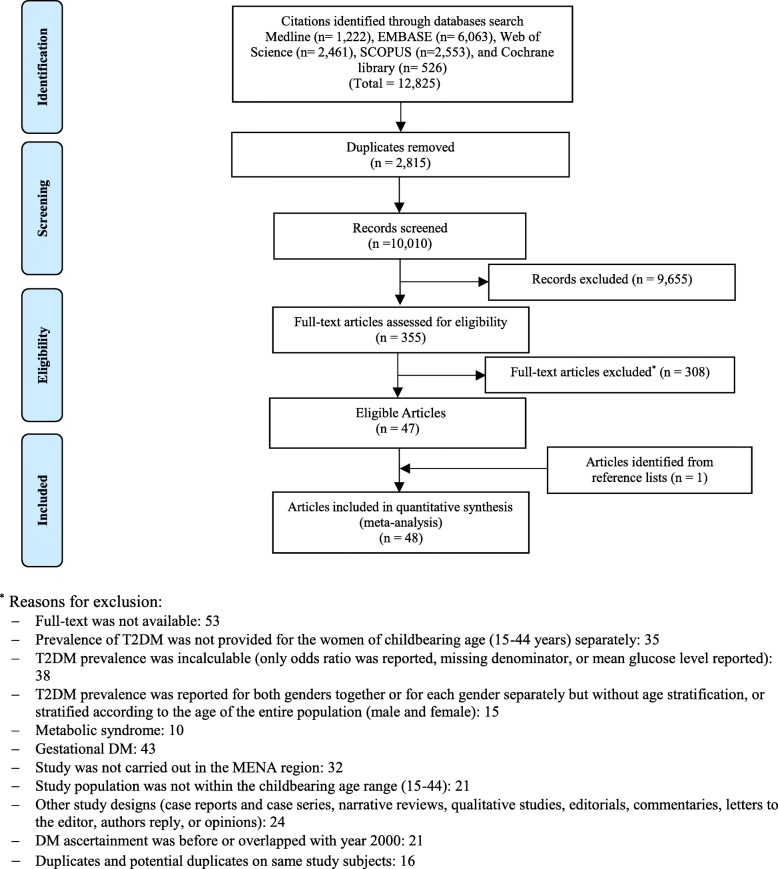
PRISMA flow chart

Detailed eligibility criteria are available in the published protocol [29]. The PRISMA flow chart for the selection of studies is shown in Fig. 1.

### Identifying eligible studies

Titles and abstracts of the remaining citations were screened independently by four reviewers (AI, KA, MM, and MQ) for any potential study on pre-DM/T2DM in childbearing age women. Full-texts of the identified potentially eligible studies were thoroughly screened and independently assessed by the four reviewers. The qualities of the extracted studies were independently assessed by two other reviewers (RHA and FA). Discrepancies in data extraction were discussed and resolved.

### Data extraction

Data from fully eligible studies were extracted into a pre-defined data extraction excel file using a pre-defined list of variables [29]. Our outcome of interest was the national/regional weighted pooled prevalence of T2DM and pre-DM in women of childbearing age in the MENA. We extracted the following data on the baseline characteristics of the eligible research reports (author names, year of publication, country, city, and study setting), study methodology (design, time period, sampling strategy, and T2DM/pre-DM ascertainment methodology), and study population (age, pregnancy status, co-morbidity, and number of women with the outcomes of interest).

In research reports which provided stratified T2DM/pre-DM prevalence estimates, the prevalence of the total sample was replaced with the stratified estimates keeping the rule of having at least 10 tested subjects per strata, otherwise we extracted information on the whole tested sample. We followed a pre-defined sequential order when extracting stratified prevalence estimates. Outcome measures stratified according to body mass index (BMI) were prioritized, followed by age and year. This prioritization scheme was used to identify the strata with more information on the tested women. When the strata were not prioritized, the overall outcome prevalence measured was extracted. For a research report that stratified the prevalence of the outcome of interest at these different levels (i.e., age and BMI), one stratum per research report was considered and included to avoid double counting. If the outcome measure was ascertained by more than one ascertainment guideline, we extracted relevant information based on the most sensitive and reliable ascertainment assay (i.e., prioritizing fasting blood glucose “FBG” over self-reported DM status), or the most recent and updated criteria (i.e., prioritizing WHO 2006 over WHO 1999 criteria).

### Meta-bias

We generated a funnel plot to explore the small-study effect on the pooled prevalence estimates. The funnel plot was created by plotting each prevalence measure against its standard error. The asymmetry of the funnel plot was tested using the Egger’s test [37] (see Additional files 3 and 4).

### Quality appraisal and risk of bias

We assessed the methodological quality and risk of bias (ROB) of the studies on T2DM or pre-DM prevalence measures using six-quality items adapted from the National Heart, Lung, and Blood Institute (NIH) tool [38]. Of the 14 items proposed for observational studies on the NIH tool, eight items were not used as they are relevant only for cohort studies assessing the relationship between an exposure and an outcome [38]. We also assessed the robustness of the implemented sampling methodology and the ascertainment methodology of the measured outcome(s) using three additional quality criteria (sampling methodology, ascertainment methodology, and precision of the estimate). Studies were considered as having “high” precision if at least 100 women tested for T2DM/pre-DM; a reasonable precision, given a pooled prevalence of 7.2% for T2DM or 7.6% for pre-DM estimated in this study, was obtained. We computed the overall proportion of research reports with potentially low risk of bias across each of the nine quality criteria. We also computed the proportion (out of nine) of quality items with potentially a low risk of bias for each of the included research reports.

### Quantitative synthesis: meta-analysis

Meta-analyses of the extracted data to estimate the weighted pooled prevalence of T2DM and pre-DM and the corresponding 95% confidence interval (CI) were executed. The variances of prevalence measures were stabilized by the Freeman-Tukey double arcsine transformation method [39, 40]. The estimated pooled prevalence measures were weighted using the inverse variance method [40], and an overall pooled prevalence estimate was generated using a Dersimonian–Laird random-effects model [41]. Heterogeneity measures were also calculated using the Cochran’s *Q* statistic and the inconsistency index; *I*–squared (*I*^2^) [42]. In addition to the pooled estimates, the prevalence measures were summarized using ranges and medians. The prediction interval, which estimated the 95% interval in which the true effect size in a new prevalence study will lie, was also reported [42, 43].

Country-level pooled estimates were generated according to the population group of tested women (general population, pregnant, non-pregnant with history of gestational DM (GDM), and patients with co-morbidity), and the overall country-level pooled prevalence, regardless of the tested population and study period. To assess if the prevalence of T2DM and pre-DM is changing over time, we stratified studies into two time periods: 2000–2009 and 2010–2018. In order not to miss any important data when estimating country-level, sub-regional, and regional prevalence, the period for studies that overlapped these two periods was defined as “overlapping”. In studies with an unclear data collection period, we used the median (~ 2 years) that was obtained from subtracting the year of publication from the year of data collection to estimate the year of data collection in those studies. The “patients with co-morbidity” included women of childbearing age with organ transplant, kidney dialysis, cancer, HIV, chronic obstructive pulmonary disease, polycystic ovarian syndrome (PCOS), or schizophrenia. Categorization of the study period was arbitrary with an aim to estimate the change in T2DM and pre-DM at the country-level and overall, over time.

We also estimated the weighted pooled prevalence, regardless of country, according to the tested women’s population group, study period, T2DM/pre-DM ascertainment guidelines (WHO guidelines, American DM Association (ADA) guidelines, International DM Association (IDF) guidelines, or medical records/anti-DM medications/self-reported), and sample size (< 100 or ≥ 100). The overall weighted pooled prevalence of T2DM and pre-DM regardless of the country, tested population, study period, ascertainment guidelines, and sample size was also generated. Providing pooled estimates regardless of the ascertainment guidelines was justified by the fact that the subject women were defined and treated as T2DM or pre-DM patients following each specific ascertainment guidelines.

To provide prevalence estimates at a more sub-regional level, countries in the MENA region were re-grouped into three sub-regions, namely, “Arab Peninsula, Fertile crescent, and North Africa and Iran.” The pooled prevalence in these three sub-regions was estimated according to the tested population group, study period, ascertainment guidelines, and sample size, as well as overall for each sub-region.

We also estimated the weighted pooled prevalence of T2DM and pre-DM according to age group. We categorized women of childbearing age into three age groups (15–29 years, 30–49 years) and not specified/overlapping. The “not specified/overlapping” category covers women who did fell in the other two age groups. For example, women with an age range of 25–34 years or 18–40 years. The age group weighted pooled prevalence produced regardless of the country, sub-region, and tested population as well as study period.

All meta-analyses were performed using the *metaprop* package [33] in Stata/SE v15 [44].

### Sources of heterogeneity: meta-regression

Random-effects univariate and multivariable meta-regression models were implemented to identify sources of between-study heterogeneity and to quantify their contribution to variability in the T2DM and pre-DM prevalence. In univariate meta-regression models, analysis was performed by country, tested population, study period, ascertainment guidelines, and sample size. All variables with a *p* < 0.1, in the univariate models, were included in the multivariable model. In the final multivariable model, a *p* value ≤ 0.05 was considered statistically significant, contributing to heterogeneity in prevalence estimates.

All meta-regression analyses were performed using the *metareg* package in Stata/SE v15 [44].

## Results

### Search and eligible research reports

Of the 12,825 citations retrieved from the six databases, 48 research reports were found eligible (Fig. 1); 46 reported T2DM prevalence [45–90] while 24 reported pre-DM prevalence [48, 49, 51–57, 60, 62, 63, 66, 67, 70, 73, 75, 81, 85, 88–90].

### Scope of reviewed T2DM reports

The 46 research reports on T2DM prevalence yielded 102 T2DM prevalence studies. The 46 reports were from 14 countries (Algeria, Egypt, Iran, Iraq, Jordan, Kuwait, Lebanon, Morocco, Oman, Qatar, Saudi Arabia, Tunisia, the United Arab Emirates [UAE], and Yemen); ranging by year between 2000 in Saudi Arabia [79] and 2018 in UAE [81]. Sixteen (34.9%) research reports were reported in Saudi Arabia [64–79], followed by 19.6% in the UAE [81–89], and 15.2% in Iran [47–53]. Over one third (37.3%) of the yielded 102 T2DM prevalence studies were in Saudi Arabia. Of the 102 T2DM prevalence studies, 79.4% were in women sampled from general populations and 11.8% in pregnant women. Over two thirds (69.6%) of the T2DM prevalence studies were in or before 2009 and 82.4% tested ≥ 100 women (Table 1).

**Table 1:** Studies reporting T2DM prevalence in childbearing age women in the MENA region, 2000–2018

Author, year [Ref]	Duration of data collection	Country, city	Setting	Design	Sampling	Population	Strata	Ascertainment method	Tested sample	Positive	Prev. (%)
Type 2 DM (46 reports in 14 countries)											
Taleb et al., 2011 [45]	1/4–3/5/2008	Algeria, Tebessa,	ANC clinics in the Protection Maternal and infant located in different parts of Tebessa	CS	Unclear	Pregnant women with an age range of 19–45 (mean age of 29.28 years)	All	Self-reported Diabetes, probably T2DM	130	3	2.3
Eldesoky et al., 2013 [46]	2009–2011	Egypt	Infertility Outpatient Clinic, Gynecology Department, Mansoura University Hospital	CS	Consecutive	Infertile young adult non-treated women with PCOS, ranging in age from 23 to 37 year.	All	Medical records	63	18	28.6
							Obese		46	15	32.6
							Lean		17	3	17.6
Ebrahimi et al., 2016 [47]	2009–2014	Iran, Shahroud	Individuals attending health centers	CS	Stratified cluster sampling method	Women attending health centers	45–49 years	First phase: DM defined as RPG ≥ 200 and/or taking antidiabetic drugs. Second phase: DM defined as FPG ≥ 6.99 mmol/L and/or A1C ≥ 6.5% and/or taking antidiabetic drugs, according to the ADA 2013 criteria	585	102	17.4
Valizadeh et al., 2015 [48]	2004–2010	Iran, Zanjan	Three main hospitals of city	RC	Whole population	Women with a history of GDM were recruited	All	DM defined as FPG levels ≥ 126 mg/dL (≥ 6.99 mmol/L) or OGTT 2-h PG ≥ 200 mg/dl	110	36	32.7
Hossein-Nezhad et al., 2009 [49]	Before 2009	Iran, Tehran	Five university educational hospitals in Tehran	CS	Consecutive	Woman gave birth (postpartum testing)	All	FBS ≥ 126 mg/dl according to the ADA criteria	2416	195	8.1
Azimi-Nezhad et al., 2009 [50]	2004	Iran, Khorasan Province	Community-based	CS	Cluster–stratified method	Iranian women from rural and urban areas in	All	DM ascertained if the subjects had a FPG ≥ 126 mg/dl (≥ 7 mmol/l) or where there was documented evidence of DM in their medical records, or treatment with hypoglycemic agents	1719	56	3.3
							15–19 years		260	12	4.6
							20–29 years		469	7	1.5
							30–39 years		465	6	1.3
							40–49 years		525	32	6.0
Azimi-Nezhad et al., 2008 [51]	Before 2008	Iran, northeast Iran	General population in urban and rural districts of the Khorasan province	CS	Multistage sampling	Women from general populations	All 15–49 years	FBS > 126 mg/dL according to the ADA 2003 guidelines	1232	40	3.2
							15–19 years		21	1	4.8
							20–29 years		258	3	1.2
							30–39 years		454	6	1.3
							40–49 years		499	30	6.0
Hadaegh et al., 2008 [52]	1999–2001	Iran, Tehran	General population. Part of Tehran Lipid and Glucose Study	CS	Multistage sampling	Women recruited from general population with mean age 43.5 years. Unclear pregnancy status.	All 20–49 years	DM defined according to ADA 2003 criteria. Undiagnosed DM: FPG < 5.6 and 2 h–PG < 7.7 mmol/L. Unknown DM: FPG 5.6 to 6.9 and 2 h–PG 7.7 to 11.0 mmol/L	3766	264	7.0
							20–29 years		1171	13	1.1
							30–39 years		1464	75	5.1
							40–49 years		1131	176	15.6
Keshavarz et al., 2005 [53]	12/1999–01/2001	Shahrood City, Iran	Fatemiyeh Hospital, Shahrood city	PS	Consecutive	All non-pregnant (postpartum) diagnosed with GDM in the recent pregnancy. Twin pregnancies, miscarriages, terminations and women with preexisting diabetes were excluded from our study	All	FPG > 126 mg/dl (7.0 mmol) on two occasions, or 2 h values in the OGTT 200 mg/dl (11.1 mmol) were diagnosed as overt diabetes according to ADA criteria	63	8	12.7
Mansour et al., 2014 [54]	1/2011–10/2012	Iraq, Basrah	Community-based	CS	Simple random	Iraqi females	All 19–45 years	According to the ADA 2010 classification: FPG ≥ 126 mg/dL (7.0 mol/L) or HbA1c ≥ 6.5% (48 mmol/mol) or OGTC 2-h plasma glucose was 200 mg/dL (11.1 mmol/L)	1332	171	12.8
							19–30 years		345	21	6.1
							31–45 years		987	150	15.2
Mansour et al., 2008 [55]	2007–2007	Iraq, Basrah	Population-based study conducted in rural areas	CS	Random sampling	Women recruited from general population with age 20–60+ years with an unclear pregnancy status	20–39 years	FPG ≥ 126 mg/dl according to the ADA 2000 criteria	148	49	33.1
Abu-Zaiton and Al-Fawwaz, 2013 [56]	10/2012–1/2013	Jordan	Al-Albayt University	CS	Random	Female university students with a mean age of 19.7 years	All	FBG > 126 mg/dL	71	2	2.8
Ahmed et al., 2013 [57]	2002–2009	Kuwait	Kuwait National Nutritional Surveillance Data	CS	Unclear	Women with age 20–69 years attending health centers for mandatory health examination for employment, pensions or Haj. Unclear pregnancy status	All 20–49 years	DM defined as FPG ≥ 7.0 mmol/L, according to the WHO 2003 criteria	2945	212	7.2
							20–29 years		1246	42	3.4
							30–39 years		857	53	6.2
							40–49 yeas		842	117	13.9
Diejomaoh et al., 2007 [58]	10/2002–06/2004	Kuwait	Obstetrics department, Maternity Hospital	CS	Consecutive	Patients who had ≥ 3 consecutive spontaneous miscarriages were classified as patients with recurrent spontaneous miscarriage	All	The fasting glucose was determined soon after the collection of the blood samples	35	0	0.0
Tohme et al., 2005 [59]	2003–2004	Lebanon	Household survey	CS	Systematic sampling	Women recruited from general population with an unclear pregnancy status	All 30–50 years	Self-reported DM. Diagnosed by a health professional and on management of DM	544	39	7.2
							30–40 years		311	16	5.1
							41–50 years		233	23	9.9
Rguibi and Belahsen, 2005 [60]	10/2001–04/2002	Morocco, Laayoune	Public Health Center during an immunization program	CS	Random Sampling	Non-pregnant women aged 15 years or older, Sahraoui ethnic origin with no previous systemic disease	All 15–34.9 years	According to the ADA criteria FPG was categorized into normal fasting glucose (NFG) (FPG,6.1 mmol/l), IFG (IFG) (FPG 6.1–6.9 mmol/ l) and diabetes (FPG > or equal 7 mmol/ l)	113	2	1.8
							15–25 years		42	0	0.0
							25–34.9 years		71	2	2.8
Gowri et al., 2011 [61]	Unclear, Over a period of one calendar year	Oman, Musact	Obstetrics Department of the Sultan Qaboos University Hospital	CS	Consecutive	Pregnant Omani women with a mean an age range of 24–42 years attending ANC care services	All	Blood testing	126	18	14.3
Al-Lawati et al., 2002 [62]	First quarter of 2000	Oman	Nation–wide survey	CS	Multi–stage stratified probability	Omani adult women ≥ 20 years	All 20–49 years	FPG ≥ 7 mmol/L according to WHO 1999 criteria or a previous history of diabetes diagnosed by a physician regardless of their FPG concentration	2088	132	6.3
							20–29 years		1186	40	3.4
							30–39 years		619	49	7.9
							40–49 years		283	43	15.2
Bener et al., 2009 [63]	1/2007–7/2008	Qatar	Population–based	CS	Multistage stratified cluster sampling	Qatari nationals above 20 years of age	All 20–49 years	FBG concentration ≥ 7.0 mmol/L and/or 2 h post–OGTT venous blood glucose concentration ≥ 11.1 mmol/L according to the WHO 2006 guidelines	471	60	12.7
							20–29 years		130	5	3.8
							30–39 years		171	14	8.2
							40–49 years		170	41	24.1
Al-Nazhan et al., 2017 [64]	2010–2012	Riyadh, Jeddah, Najran, Albaha	Dental Clinics in the cities and King Saud University Riyadh	CS	Random	The samples were randomly selected according to the following inclusions criteria: subjects over 16 years of age with more than 10 teeth (excluding third molars) who required the panoramic radiograph as part of dental diagnosis and treatment plan were included in the study	All 16–45 years	Unclear	295	3	1.0
							16–25 years		121	1	0.8
							26–35 years		110	0	0.0
							36–45 years		64	2	3.1
Saeed, 2017 [65]	2005	Saudi Arabia	Primary Health Centers	CS	Multistage stratified cluster random sampling	Saudi adults aged 15–64 years	All 15–49 years	Data was collected using the WHO STEP-wise questionnaire which includes sociodemographic, life style habits, NCD, associated factors in addition to biochemical and blood pressure measurements	1854	322	17.4
							15–24 years		464	32	6.9
							25–34 years		573	65	11.3
							35–44 years		598	148	24.7
							45–49 years		219	77	35.2
Bahijri et al., 2016 [66]	Unclear	Saudi Arabia, Jeddah	Household survey	CS	Multistage sampling	Women recruited from general population, with age 18–60+ years with an unclear pregnancy status	All	FPG ≥ 126 mg/dl and/or HbA1c ≥ 6.5%	608	39	6.4
							18–20 years		126	0	0.0
							20–< 30 years		183	4	2.8
							30–< 40 years		153	8	6.5
							40–< 50 years		146	27	18.5
Al-Rubeaan et al., 2015 [67]	2007–2009	Saudi Arabia, 13 regions	Saudi–DM national level household population-based study	CS	Random sampling	Men & Women with age 30 – ≥ 70 years with known & unknown GDM and DM status	All 30–49 years	DM defined according to ADA 2011 criteria (FPG ≥ 126 mg/dL)	285	35	12.3
							30–39 years		212	20	9.4
							40–49 years		73	15	21
Al Serehi et al., 2015 [68]	2011–2013	Saudi Arabia, Riyadh	A single center study conducted at King Fahad Medical City	CS	Whole population	Pregnant women with mean age 29.9 years	All	Medical records and unclear	1718	14	0.9
Al-Rubeaan et al., 2014 [69]	2007–2009	Saudi Arabia, Nationwide	Saudi–DM national level household population-based study	CS	Random sampling	Pregnant women in different trimesters, recruited from general population with age 18–49 years	All 18–49 years	FPG according to ADA 2011 criteria and self-reported	549	16	2.9
							18–29 years		264	3	1.1
							30–39 years		212	7	3.3
							40–49 years		73	6	8.2
Amin et al., 2014 [70]	2012–2012	Saudi Arabia, Al-Hassa	Primary care center located in King Faisal University	CS	Unclear	Non-pregnant university employees with age 20–63 years	All 20–49 years	FPG ≥ 126 mg/dl and/or using antidiabetic medicines	166	11	6.6
							20–< 30 years		31	0	0.0
							30–< 40 years		68	3	4.4
							40–< 49 years		67	8	11.9
Wahabi et al., 2012 [71]	1/1/−31/12/2008	Saudi Arabia, Riyadh	King Khalid University Hospital	CS	Unclear	Women who were admitted to the labor ward in King Khalid University Hospital	All	Medical records where DM was ascertained before the index of pregnancy	3157	50	1.6
Saeed 2012 [72]	2005–2005	Saudi Arabia, Riyadh	National population-based survey conducted in 20 health regions	CS	Multistage sampling	Women recruited from general population with age 15–64 years. Unclear pregnancy status.	All 15–44 years	Self-reported DM. Diagnosed by a health professional and on management of DM	1659	131	7.9
							15–24 years		528	12	2.3
							25–34 years		556	27	4.8
							34–44 years		575	92	16.0
Al-Daghri et al., 2011 [73]	Unclear	Saudi Arabia, Riyadh	Patients recruited from homes and invited to visit primary healthcare centers	CS	Random sampling	Women attending outpatient clinics with age 7–80 years. Unclear pregnancy status	18–45 years	According to WHO 1999 criteria. FPG ≥ 7.0 mmol/L	2373	228	9.6
Alqurashi et al., 2011 [74]	6/2009	Saudi Arabia, Riyadh	King Fahad Armed Forces Hospital	CS	All patients during the study period	Female patients attending a primary care clinic	All 20–49 years	Self-reported confirmed by diabetic therapies	2361	343	14.5
							20–29 years		942	44	4.7
							30–39 years		761	82	10.8
							40–49 years		658	217	33.0
Al-Baghli et al., 2010 [75]	28/8/2004–18/2/2005	Saudi Arabia	Community-based	CS	All residents were invited to participate in this survey	Saudi female subjects aged 30 years and above who resided in the Eastern Province	All 30–49 years	History of DM or FBG of ≥ 126 mg/dl (≥ 7.0 mmol/l), or the casual capillary blood glucose was ≥ 200 mg/dl (≥ 11.0 mmol/l) according to the ADA 2003 guidelines	9092	1525	16.8
							30–39 years		2870	175	6.1
							40–49 years		6222	1350	21.7
Al-Qahtani et al., 2006 [76]	2004–2005	Saudi Arabia, King Khalid Military City, Northern	Primary Health Clinics	CS	Whole population	Non-Pregnant women	All 18–49 years	Individuals with self-reported history of DM with anti-DM medication and those with FPG ≥ 7 mmol/L	1906	94	5.1
							18–19 years		83	0	0.0
							20–29 years		737	13	1.8
							30–39 years		890	45	5.1
							40–49 years		196	36	18.4
Shaaban et al., 2006 [77]	2001–2002	Saudi Arabia, Jeddah	Maternity and Children’s Hospital	CS	Consecutive	All pregnant women with a singleton live birth	All	Unclear	313	10	3.2
						All pregnant women admitted to the hospital with a diagnosis of singleton IUFD at the third trimester with a fetal weight of 1500 g and more. Multiple pregnancy and intra-partum IUFD were excluded			157	41	26.1
Habib, 2002 [78]	2000	Saudi Arabia, Riyadh	Obstetrics Unit King Khalid University Hospital	CS	Consecutive	Pregnant women undergone Cesarean section during the time period of the study in the hospital	< 20–40+ years	Medical records	754	136	18.0
Karim et al., 2000 [79]	Unclear	Saudi Arabia, Riyadh	Al-Kharj Military Hospital	CS	Random selection of medical records	Female patients age 18–34 years	All	Medical records	599	4	0.7
Ben Romdhane et al., 2014 [80]	2005	Tunisia	National household survey	CS	Multistage sampling	Women recruited from general population, with age 35–65+ years with an unclear pregnancy status	All	According to WHO 1999 criteria. FPG ≥ 6.1, or confirmed or self-reported use of anti-DM medications in the past 2 weeks	2191	190	8.7
							35–39 years		709	36	5.1
							40–44 years		758	67	8.9
							45–49 years		724	87	12.0
Sulaiman et al., 2018 [81]	2013–2014	UAE, (Dubai, Sharjah and Northern Emirates)	Preventive Medicine Departments (Visa Renewal Screening Centers)	CS	Systematic Random Sampling	Migrants recruited from Visa Renewal Centers	All 18–50 years	Medically confirmed DM and were either using glucose-lowering medications or had a FPG ≥ 7.0 mmol/L or HbA1c ≥ 6.5% were classified as with known DM	429	24	5.6
							18–30 years		133	6	4.5
							31–40 years		198	11	5.6
							41–50 years		98	7	7.1
							All 18–50 years	FPG or HbA1c levels within the diabetes range. Cut-off values were used according to ADA to diagnose New DM cases	429	22	5.1
							18–30 years		133	6	4.5
							31–40 years		198	6	3
							41–50 years		98	10	10.2
Shah et al., 2017 [82]	2012–2013	UAE, Al Ain	Visa screening center	CS	Systematic sampling	Migrant workers with mean age 34.1 years with an unclear pregnancy status	18–40 years	Self-reported, or use of a diabetic medication or HbA1c ≥ 6.5%, according to the ADA 2015 criteria	156	19	12.2
Al Dhaheri et al., 2016 [83]	2013–2014	UAE, Al Ain	UAE University	CS	Random sampling	University students with age 17–25 years. Unclear pregnancy status	All	Self-reported DM. Impaired fasting glucose ≥ 100 mg/dl or use of hypoglycemic medicines, following the IDF, AHA/NHLBI criteria	555	54	9.7
Agarwal et al., 2015 [84]	1/1/2012–31/12/2012	UAE, Al Ain	Tawam Hospital	CS	Unclear	Pregnant women attending the routine ANC clinics who were unaware of their antepartum DM status	All	FPG and/or 2–h OGTT glucose ≥ 7.0 mmol/L and 11.1 mmol/L based on cut-off values of the ADA cut-off point, according to the ADA 2003 criteria	2337	50	2.1
Hajat et al., 2012 [85]	2008–2010	UAE, Abu Dhabi	SEHA primary healthcare centers in Abu Dhabi Emirate	CS	Whole population	Women enrolled in Waqaya screening program with age 18–75 years. Pregnancy status not reported	All 18–49 years	DM defined as taking diabetes medicines, HbA1c ≥ 6.5%, or random blood glucose > 11.1 mmol/L, following the ADA 2010 criteria	21,792	1774	8.1
							18–20 years		250	3	1.2
							20–29 years		10,629	287	2.7
							30–39 years		7216	534	7.4
							40–49 years		3697	950	25.7
Baynouna et al., 2008 [86]	3/2004–2/2005	UAE, Al Ain	Community-based	CS	Stratified random sampling	UAE citizens with mean age 44.1 years. Unclear pregnancy status	All 20–49 years	Following ADA 2005 guidelines. FPG > 125 mg/dl, patient using diabetic medications or self-reported diabetes	299	40	13.4
							20–29 years		59	1	1.7
							30–39 years		114	6	5.3
							40–49 years		126	33	26.2
Saadi et al., 2007 [87]	12/2005–11/2006	UAE, Al Ain	Population–based	CS	Random	Non-pregnant UAE citizen women ≥ 18 years	All 18–49 years	FBG concentration ≥ 7.0 mmol/L and/or 2 h post–OGTT venous blood glucose concentration ≥ 11.1 mmol/L according to the WHO 1999 guidelines	1028	108	10.5
							18–29 years		627	62	1.0
							30–39 years		240	16	6.7
							40–49 years		161	30	18.6
Malik et al., 2005 [88]	10/1999–06/2000	UAE	Population-based study	CS	Stratified, multistage, random sample	Non-institutionalized subjects residing in the UAE and aged 20 years and above. The study design included only people who were living in a family residence and sharing the same income and excluded anyone who lived in workers barracks	All 20–44 years	FBG ≥ 7.0 mmol/L or greater than, 7.0 mmol/L and/or 2-h venous blood glucose concentration equal to or greater than, 11.1 mmol/l, or currently on hypoglycaemic agents. Abnormal glucose tolerance defined according to the latest recommendations of a WHO expert group	2355	296	12.6
							20–24 years		339	4	1.2
							25–34 years		862	72	8.4
							35–44 years		1154	220	19.1
Agarwal et al., 2004 [89]	1/1998–12/2002	UAE, Al Ain	Obstetric clinics at the Al Ain Hospital	RS	Unclear	Women diagnosed with GDM who had the 2 h, 75 g OGTT, 4–8 weeks after delivery with a mean maternal age of 32 years	All	FPG ≥ 7 mmol/L and/or 2 h PG ≥ 11.1 mmol/L according to the WHO 1999 guidelines	549	50	9.1
Gunaid and Assabri, 2008 [90]	Unclear	Yemen, Sana’a	Semirural area of Hamdan	CS	Multistage random sampling	Women with an age range of 35–44 years	All	T2DM defined as 2-h capillary whole blood glucose concentration ≥ 11.1 mmol/L according to the WHO 1999 guidelines	54	5	9.3
Pre-DM (*24 reports in 10 countries*)											
Valizadeh et al., 2015 [48]	2004–2010	Iran, Zanjan	Three main hospitals of city	RC	Whole population	Women with a history of GDM	All	IFG defined as FBS between 100 and 126 mg/dL (5.5–6.99 mmol/L)	110	1	0.9
								IGT defined as blood sugar level of 140 to 199 mg/dL (7.77–11.04 mmol/L) in OGTT		10	9.1
Hossein-Nezhad et al., 2009 [49]	Before 2009	Iran, Tehran	Five university educational hospitals in Tehran	CS	Consecutive	Woman gave birth with history of GDM (postpartum testing)	IGT	2-h postprandial glucose between 140 mg/dl and 199 mg/dl (7.8–11.0 mmol/l), according to the ADA criteria	2416	517	21.4
Hadaegh et al., 2008 [52]	1999–2001	Iran, Tehran	General population. Part of Tehran Lipid and Glucose Study.	CS	Multistage sampling	Women recruited from general population with mean age 43.5 years with an unclear pregnancy status	IFG/All 20–49 years	IFG defined according to the 2003 ADA criteria. IFG 5.6 to 6.9 and 2 h–PG < 7.7 mmol/L	3766	207	5.6
							20–29 years		1171	40	3.4
							30–39 years		1464	79	5.4
							40–49 years		1131	88	7.8
Azimi-Nezhad et al., 2008 [51]	Before 2008	Iran, northeast Iran	Genral population in urban and rural districts of the Khorasan province	CS	Multistage sampling method	Women from general populations	IFG/All	FBS between 110 and 126 mg/dL, according to the ADA 2003 criteria	1232	15	1.2
							15–19 years		21	0	0.0
							20–29 years		258	1	0.4
							30–39 years		454	4	0.9
							40–49 years		499	10	2.0
Hossein-Nezhad et al., 2007	Before 2007	Iran, Tehran	Five teaching hospitals affiliated to Tehran University of Medical Sciences	CS	Consecutive	Pregnant women referred to ANC visits with no known history of diabetes	IGT/All	According to the Carpenter and Coustan 1979 criteria	2416	70	2.9
							15–24 years		1209	21	1.7
							25–34 years		1001	39	3.9
							35–45 years		206	10	4.9
Keshavarz et al., 2005 [53]	12/1999–01/2001	Iran, Shahrood City	Fatemiyeh Hospital, Shahrood city	PS	Consecutive	All non-pregnant (postpartum) diagnosed with GDM in the recent pregnancy. Excluding twin pregnancies, miscarriages, terminations and preexisting DM	All	Non-diabetic individuals with an FPG > 110 mg/dl (6.1 mmol) but < 126 mg/dl were considered to have IFG and those with 2 h value in the OGTT 140 mg/dl (7.8 mmol) but < 200 mg/dl were defined as IGT, based on ADA criteria	63	7	11
Mansour et al., 2014 [54]	1/2011–10/2012	Iraq, Basrah	Community-based	CS	Simple random	Iraqi females with an age range of 19–94 years	IFG (All)	According to the ADA 2010 classification: FPG ranging from 100 mg/dL (5.6 mmol/L) to 125 mg/dL (6.9 mmol/L) or HbA1c ranging from 5.7% (39 mmol/mol) to 6.4% (46 mmol/mol) or OGCT with plasma glucose lvele of 140–199 mg/dL (7.7–11 mmol/L)	1332	324	24.3
							19–30 years		345	54	15.6
							31–45 years		987	270	27.4
Mansour et al., 2008 [55]	2007–2007	Iraq, Basrah	Population-based study conducted in rural areas.	CS	Random sampling	Women recruited from general population with age 20–60+ years. Unclear pregnancy status	All 20–39 years	IFG defined as FPG 100–125 mg/dl, according to the ADA 2000 criteria	40	16	40.0
Abu-Zaiton and Al-Fawwaz, 2013 [56]	10/2012–1/2013	Jordan	Al-Albayt University	CS	Random sampling	Female university students with a mean age of 19.71 years ±2.55 SD	IFG	FBG between 100 and 126 mg/dL	71	10	14.1
Ahmed et al., 2013 [57]	2002–2009	Kuwait	Kuwait National Nutritional Surveillance Data collected from primary health centers	CS	Unclear	Women with age 20–69 years attending health centers for mandatory health examination for employment, pensions or Haj. Unclear pregnancy status	IFG/All 20–49 years	FBG between 6.1 and 6.9 mmol/L, according to the WHO 2003 criteria	2945	278	9.4
							20–29 years		1246	102	8.2
							30–39 years		857	76	8.9
							40–49 years		842	100	11.9
Alattar et al., 2012	2009–2010	Kuwait	College of applied education and training	CS	Unclear	Non-pregnant college students with mean age 20.3 years	17–24 years	IGR/IGT defined as the presence of one or more of the following: FPG of ≥ 5.6 to < 7 mmol/l, 2-h postprandial glucose level of ≥ 7.8 to < 11.1 mmol/L and HbA 1c ≥ 5.6 to < 6.5%, according to the ADA 2010 criteria	311	96	31
Rguibi and Belahsen, 2005 [60]	2001–2002	Morocco, Laayoune	Public health centers	CS	Random sampling	Non-pregnant women with mean age 36.8 years visiting health centers during an immunization campaign	IFG/All 15–34.9 years	FPG between 6.1–6.9 mmol/L, following the ADA 1997 criteria	113	1	0.9
							15–25 years		42	0	0.0
							25–34.9 years		71	1	2.8
Al-Lawati et al., 2002 [62]	First quarter of 2000	Oman	Nation–wide survey	CS	Multi–stage stratified probability	Adult women of Omani nationals aged ≥ 20 years	IFG (All)	FPG ≥ 6.1 but < 7 mmol/ l, according to the WHO 1999 criteria	2088	82	3.9
							20–29 years		1186	28	2.4
							30–39 years		619	37	6.0
							40–49 years		283	17	6.0
							40–49 years		170	4	2.4
							IGT/All	IGR/IGT defined according to WHO criteria. 2 h post–OGTT PG 7.8–11.0 mmol/L, according to WHO 2006 criteria	471	60	12.7
							20–29 years		130	10	7.7
							30–39 years		171	22	12.9
							40–49 years		170	28	16.5
Bener et al., 2009 [63]	1/2007–7/2008	Qatar	Population–based	CS	Multistage stratified cluster	Qatari nationals above 20 years of age	IFG/All	FBG between 5.6 and 6.9 mmol/l, according to the WHO 2006 criteria	471	4	0.8
							20–29 years		130	0	0.0
							30–39 years		171	0	0.0
							40–49 years		170	4	2.4
Bahijri et al., 2016 [66]	Before 2016	Saudi Arabia, Jeddah	Household survey	CS	Multistage sampling	Women recruited from general population with age 18–> 60 years. Unclear pregnancy status	All 18–49 years	FBG of 100–125 mg/dl and/or HbA1c 5.7–6.4%	608	40	6.6
							18–20 years		126	3	2.4
							20–< 30 years		183	6	3.3
							30–< 40 years		153	15	9.8
							40–< 50 years		146	16	11.0
Al-Rubeaan et al., 2015 [67]	2007–2009	Saudi Arabia, 13 regions	SAUDI–DM national level population-based study.	CS	Unclear	Men & Women with age 30 – ≥ 70 years with known & unknown GDM and DM status	All 30–49 years	FBG between 5.6 and 6.9 mmol/L, according to the ADA 2011criteria	285	61	21.4
							30–39 years		212	42	19.8
							40–49 years		73	19	26.3
Amin et al., 2014 [70]	2012–2012	Saudi Arabia, Al-Hassa	Primary care center located in King Faisal University	CS	Unclear	Non-pregnant university employees with age 20–63 years	IFG/All 20–49 years	FBG between 110 and 125 mg/dl	166	9	5.4
							20–< 30 years		31	0	0.0
							30–< 40 years		68	3	4.4
							40–< 49 years		67	6	9.0
Al-Daghri et al., 2011 [73]	Before 2011	Saudi Arabia, Riyadh	Patients recruited from homes and invited to visit primary healthcare centers.	CS	Random sampling	Women attending outpatient clinics with age 7–80 years. Unclear pregnancy status	All 18–45 years	FPG between 6.1 and 6.9 mmol/L (110 to 125 mg/dL), according to the WHO 1999 criteria	2373	204	8.6
Al-Baghli et al., 2010 [75]	28/8/2004–18/2/2005	Saudi Arabia	Community-based	CS	All residents were invited to participate in this survey	Saudi female subjects aged 30 years and above who resided in the Eastern Province	IFG (All)	FPG between 100 and 125 mg/dl (5.6–6.9 mmol/l), according to the ADA 2003 criteria	1971	50	2.5
							30–39 years		783	13	1.7
							40–49 years		1188	47	4.1
Sulaiman et al., 2018 [81]	2013–2014	Dubai, Sharjah, Northern Emirates (UAE)	UAEDIAB UAE National Diabetes and Lifestyle Population-b ased	CS	Systematic Random Sampling	A random sample of migrants recruited from Visa renewal Centers	IFG/All 18–50 years	FPG between 6.1 and 6.9 mmol/L	429	52	12.1
							18–30 years		133	12	9.1
							31–40 years		198	25	12.7
							41–50 years		98	15	15.3
Hajat et al., 2012 [85]	2008–2010	UAE, Abu Dhabi	SEHA primary healthcare centers in Abu Dhabi Emirate	CS	Whole population	Women with age 18–75 years. Unclear pregnancy status	All 18–49 years	HbA1c 5.7–6.4%, according to the ADA 2010 criteria	21,940	5844	26.6
							18–20 years		244	49	20.1
							20–29 years		10,785	2362	21.9
							30–39 years		7220	2130	29.5
							40–49 years		3691	1303	35.3
Malik et al., 2005 [88]	10/1999–06/2000	UAE	Population-based study	CS	Stratified, multistage, random sample	Only people (≥ 20 years) who were living in a family residence and sharing the same income and excluded anyone who lived in workers barracks	IFG/All 20–44 years	FBG between 6.1 and 6.9 mmol/l	2355	175	7.4
							20–24 years		339	17	5.0
							25–34 years		862	58	6.7
							35–44 years		1154	100	8.7
						Only people (≥ 20 years) who were living in a family residence and sharing the same income and excluded anyone who lived in workers barracks	IGT/All 20–44 years	2 h venous blood glucose level of 7.8–11.0 mmol/L on the OGT test	2059	347	16.8
							20–24 years		335	43	12.7
							25–34 years		790	113	14.3
							35–44 years		934	191	20.5
Agarwal et al., 2004 [89]	1/1998–12/2002	UAE, Al Ain	Obstetric clinics at the Al Ain Hospital	RS	Unclear	Women diagnosed with GDM who had the 2 h, 75 g OGTT, 4–8 weeks after delivery with a mean maternal age of 32 years	IGT	WHO 1999 criteria: FPG < 7 mmol/L and 2 h PG, 7.8–11.0 mmol/l	549	84	15.3
							IFG	WHO 1999 criteria: FPG 6.1–6.9 mmol/l	549	30	5.5
Gunaid and Assabri, 2008 [90]	Unclear	Yemen, Sana’a	Semirural area of Hamdan	CS	Multistage random technique was used	Women with an age range of 35–44 years	IGT	IGT < 6.1 mmol/L and 2–h capillary whole blood glucose concentration from ≥ 7.8 mmol/L to < 11.1 mmol/L, according to the WHO 1999 criteria	54	4	7.4
							IFG	FBG between 5.6 and 6.1 mmol/L, and 2-h capillary whole blood glucose concentration < 7.8 mmol/L, according to the WHO 1999 criteria	54	2	3.7

*CS* cross-sectional, *RS* retrospective, *PS* prospective, *GCT* glucose challenge test, *OGTT* oral glucose tolerance test, *DM* diabetes mellitus, *T2DM* type 2 diabetes, *GDM* gestational diabetes. *ADA* American Diabetes Association, *WHO* World Health Organization, *UAE* United Arab Emirates, *FB/PG* fasting blood/plasma glucose, *FB/PS* fasting blood/plasma sugar, *RPG* random plasma glucose, *PCOS* polycystic ovary syndrome, *ANC* antenatal care, *IUFD* intrauterine fetal death; *WHO STEP* WHO STEP-wise approach to surveillance, *HbA1c* glycosylated hemoglobin, *IFG* impaired fasting glucose, *IGT* impaired glucose tolerance, *IDF*: International Diabetes Federation

#### Pooled T2DM prevalence

**Fig. 2:**
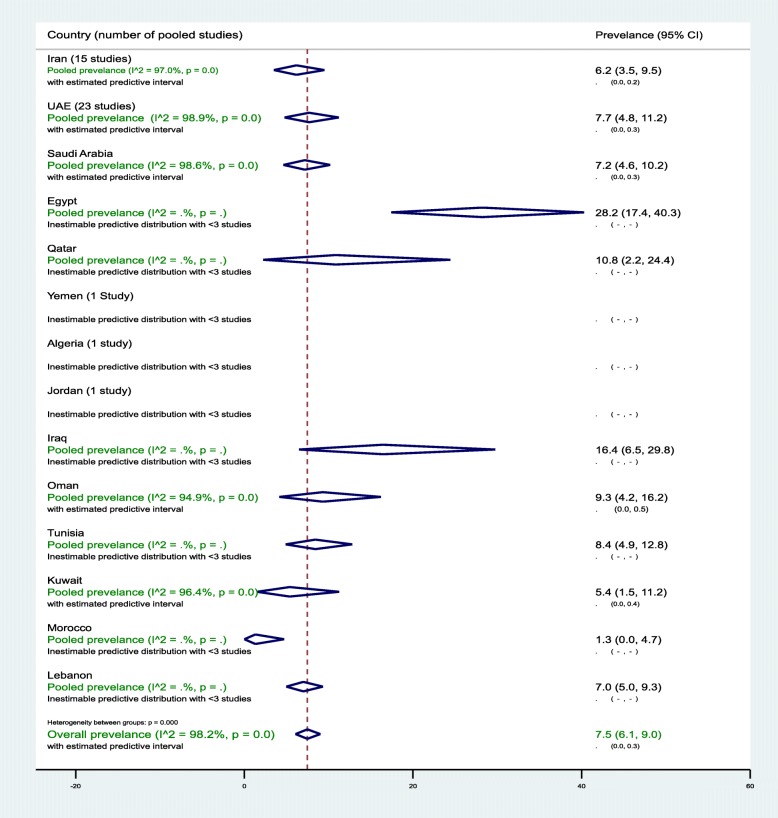
Forest plot of the meta-analyses for the 14 MENA countries’ studies on T2DM Pooled findings of 102 T2DM prevalence estimates reported in 14 countries in the MENA region. The individual 102 estimates and their 95% confidence interval (CI) omitted to fit the plot. The diamond is centered on the summary effect estimate, and the width indicates the corresponding 95% CI. UAE, United Arab Emirates; T2DM, type 2 diabetes mellitus; MENA, Middle East and Northern Africa

In the 14 countries, the weighted T2DM prevalence in women of childbearing age estimated at 7.5% (95% CI, 6.1–9.0%, *I*^2^*,* 98.2%) (Table 2, Fig. 2). The weighted T2DM prevalence was not significantly different (*p* = 0.4) in studies reported between 2000 and 2009 (7.9%, 95% CI, 6.2–9.7%, *I*^2^, 97.9%) and studies reported between 2010 and 2018 (5.8%, 95% CI, 3.4–8.7%, *I*^2^, 95.4%) (Table 2). The weighted T2DM prevalence was higher in women with an age range of 15–19 years (10.9%, 95% CI, 8.8–13.3%, *I*^2^, 97.9%) than women with an age range of 30–49 years (2.5%, 95% CI, 1.8–3.2%, *I*^2^, 83.6%) (see Additional file 5).

**Table 2:** Weighted national prevalence of T2DM in childbearing age women in MENA countries

Country/population	No. of studies	Tested sample	T2DM	T2DM prevalence					Heterogeneity measures		
				Range (%)	Median (%)	Weighted prev. %	95% CI	Subgroup *p* value	*Q* (*p* value)^1^	*I*^2^ (%)^2^	95% prediction interval (%)^3^
Algeria											
Pregnant	1	130	3	2.3	NE	NE	NE	NE	NE	NE	NE
Egypt											
Infertile	2	63	18	17.6–32.6	25.1	28.2	17.4–40.3	NE	NE	NE	NE
Iran											
General population^4^	8	11,143	406	1.1–17.4	4.9	5.3	1.7–10.6	< 0.001	334.2 (*p* < 0.001)	97.9	0.0–30.0
Pregnant	5	4135	252	1.3–8.1	4.6	3.9	1.5–7.4		75.0 (*p* < 0.001)	94.7	0.0–20.0
Non-pregnant with a history of GDM	2	173	44	12.7–32.7	22.7	24.7	18.5–31.5		NE	NE	NE
Study period^5^											
2000–2009	13	14,324	564	1.1–15.6	4.8	4.3	2.2–7.0	< 0.001	328.4 (*p* < 0.001)	96.3	0.0–20.0
2010–2018	1	1170	102	17.4	NE	NE	NE		NE	NE	NE
Overlapping^6^	1	110	36	32.7	NE	NE	NE		NE	NE	NE
Overall^7^	15	15,604	702	1.1–32.7	5.1	6.2	3.5–9.5		471.6 (*p* < 0.001)	97.0	0.0–20.0
Iraq											
General population^4^	3	1480	220	6.1–33.1	15.2	16.4	6.5–29.8	NE	56.7 (*p* < 0.001)	96.5	NE
Study period^5^											
2000–2009	1	148	49	33.1	NE	NE	NE	< 0.001	NE	NE	NE
2010–2018	2	1332	171	6.1–15.2	10.6	12.5	10.8–14.3		NE	NE	NE
Jordan											
General population^4^ (2012–2013)	1	71	2	2.8	NE	NE	NE	NE	NE	NE	NE
Kuwait											
General population^4^ (2002–2009/2004)	4	2980	212	0.0–13.9	4.8	5.4	1.5–11.2	NE	82.8 (*p* < 0.001)	96.4	0.0–40.0
Lebanon											
General population^4^ (2003–2004)	2	544	39	5.1–9.9	7.5	7.0	5.0–9.3	NE	11.2 (*p* < 0.001)	NE	NE
Morocco											
General population^4^ (2001–2002)	2	113	2	0.0–2.8	1.4	1.3	0.0–4.7	NE	1.6 (*p* = 0.1)	NE	NE
Oman											
General population^4^ (first quarter of 2000)	3	2088	132	3.4–15.2	7.9	8.0	2.9–15.4	0.2	48.9 (*p* < 0.001)	95.9	NE
Pregnant (before 2011)	1	126	18	14.3	NE	NE	NE		NE	NE	NE
Overall^7^	4	2214	140	3.4–15.2	11.1	9.3	4.2–16.2		59.1 (*p* < 0.001)	94.9	0.0–50.0
Qatar											
General population^4^ (2007–2008)	3	471	60	3.8–24.1	8.2	10.8	2.2–24.4	NE	31.5 (*p* < 0.001)	93.7	NE
Saudi Arabia											
General population^4^	30	21,452	2748	0.0–35.2	6.5	8.0	5.3–11.3	< 0.001	1679.1 (*p* < 0.001)	98.3	0.0–30.0
Pregnant	4	5942	210	0.8–18.0	2.4	4.3	0.5–11.5		281.7 (*p* < 0.001)	98.9	0.0–60.0
Patients^8^	4	452	44	0.0–26.1	0.8	4.5	0.0–19.9		80.7 (*p* < 0.001)	96.3	0.0–10.0
Study period^5^											
2000–2009	27	25,059	2935	0.0–35.2	8.2	9.2	6.0–13.0	< 0.001	2214 (*p* < 0.001)	98.8	0.0–40.0
2010–2018	11	2787	67	0.0–18.5	2.2	2.8	0.7–6.0		101.7 (*p* < 0.001)	90.2	0.0–20.0
Overall^7^	38	27,846	3002	0.0–35.2	5.1	7.2	4.6–10.2		2679.8 (*p* < 0.001)	98.6	0.0–30.0
Tunisia											
General population^4^ (2005)	3	2191	190	5.1–12.0	8.8	8.4	4.9–12.8	NE	23.0 (*p* < 0.001)	91.3	NE
United Arab Emirates											
General population^4^	21	27,043	2337	1.2–26.2	7.1	8.0	4.8–11.9	< 0.001	1777.5 (*p* < 0.001)	98.9	0.0–30.0
Pregnant	1	2337	50	2.1	NE	NE	NE		NE	NE	NE
Non-pregnant with a history of GDM	1	549	50	9.1	NE	NE	NE		NE	NE	NE
Study period^5^											
2000–2009	10	4231	494	1.2–26.2	8.7	9.4	5.6–14.1	0.4	182.9 (*p* < 0.001)	95.1	0.0–30.0
2010–2018	9	3906	169	2.1–12.2	5.6	6.0	3.3–9.5		84.5 (*p* < 0.001)	90.5	0.0–20.0
Overlapping^6^	4	21,792	1774	1.2–25.7	5.1	7.3	1.2–17.9		1499.9 (*p* < 0.001)	99.8	0.0–80.0
Overall^7^	23	29,929	2437	1.2–26.2	7.1	7.7	4.8–11.2		1921.1 (*p* < 0.001)	98.9	0.0–30.0
Yemen											
General population^4^ (Before 2011)	1	54	5	9.3	NE	NE	NE	NE	NE	NE	NE
All countries											
Population											
General population^5^	81	69,630	6353	0.0–35.2	6.2	7.7	6.1–9.4	< 0.001	4443.5 (*p* < 0.001)	98.2	0.0–30.0
Pregnant	12	12,670	533	0.8–18.0	2.8	4.3	2.1–7.0		460.7 (*p* < 0.001)	97.6	0.0–20.0
Non-pregnant with a history of GDM	3	722	94	9.1–32.7	12.7	17.0	4.9–34.1		NE	94.1	NE
Patients^8^	4	605	44	0.0–26.1	2.0	4.5	0.0–19.9		NE	44.8	NE
Infertile	2	63	18	17.6–32.6	25.1	28.2	17.4–40.3		NE	NE	NE
Study period^5^											
2000–2009	71	52,459	4703	0.0–35.2	6.7	7.9	6.2–9.7	0.4	3280.9 (*p* < 0.001)	97.9	0.0–30.0
2010–2018	26	9329	529	0.0–32.6	4.9	5.8	3.4–8.7		547.5 (*p* < 0.001)	95.4	0.0–30.0
Overlapping^6^	5	21,902	1810	1.2–32.7	7.4	10.9	3.4–21.8		1553.0 (*p* < 0.001)	99.7	0.0–60.0
Ascertainment^9^											
WHO guidelines	27	14,843	1358	0.0–32.7	8.4	8.6	6.6–10.8	0.4	510.8 (*p* < 0.001)	94.9	0.0–20.0
ADA guidelines	34	50,307	4312	0.0–33.1	6.0	7.4	4.9–10.3		3315.2 (*p* < 0.001)	99.7	0.0–30.0
IDF guidelines	1	555	54	9.7	9.7	NE	NE		NE	NE	NE
Medical records/anti-DM medications/self-reported	40	17,985	1318	0.0–35.2	5.0	6.8	4.5–9.5		1595.7 (*p* < 0.001)	97.6	0.0–30.0
Sample size											
< 100	16	1002	72	0.0–32.1	4.8	5.5	2.7–9.1	0.4	69.7 (*p* < 0.001)	75.6	0.0–20.0
≥ 100	84	82,688	6970	0.0–35.2	6.1	7.8	6.3–9.5		5513.1 (*p* < 0.001)	98.5	0.0–30.0
Overall^10^	102	83,690	7042	0.0–35.2	6.1	7.5	6.1–9.0		5583.0 (*p* < 0.001)	98.2	0.0–30.0

^1^*Q*: Cochran’s *Q* statistic is a measure assessing the existence of heterogeneity in estimates of T2DM prevalence

^2^*I*^2^: a measure assessing the percentage of between-study variation that is due to differences in T2DM prevalence estimates across studies rather than chance

^3^Prediction interval: estimates the 95% confidence interval in which the true T2DM prevalence estimate in a new study is expected to fall

^4^General populations could include healthy population, health care workers, migrant workers, or employees

^5^Year range does not cover every single year within that range. In studies with unclear information on when the study was conducted, we subtracted 2 years from the publication year as this was the median of the data collection period and the publication year for the other studies with full information

^6^Study period was before and after 2009

^7^Pooled estimate, regardless of the tested population, sample size, and data collection period, used the most updated criteria when T2DM was ascertained, based on different criteria in the same population

^8^Patients could be those on kidney dialysis, or with arthritis, organ transplant, cancer, HIV, COPD, PCOS, or schizophrenia

^9^Regardless of the year of the guidelines for the most updated criteria when T2DM was ascertained, based on different criteria in the same population

^10^Overall pooled estimate in the 15 countries regardless of the tested population, sample size, and data collection period, using the most updated criteria when T2DM ascertained using different criteria in the same population

*NE* not estimable, *CI* confidence interval calculated using the exact binomial method, *T2DM* type 2 diabetes mellitus, *GDM* gestational diabetes, *WHO* World Health Organization, *ADA* American Diabetes Association, *IDF* International Diabetes Federation, *HIV* human immunodeficiency syndrome, *COPD* chronic obstructive pulmonary disease, *PCOS* polycystic ovary syndrome

The highest two weighted T2DM estimates were observed in infertile women of childbearing age in Egypt (28.2%, 95% CI, 17.4–40.3%) and in non-pregnant women with a history of GDM in Iran (24.7%, 95% CI, 18.5–31.5%) (Table 2). In general populations, the weighted T2DM prevalence ranged between 1.3% (95% CI, 0.0–4.7%) in 2001–2002 in Morocco [60] and 16.4% (95% CI, 6.5–29.8%, *I*^*2*^, 96.5%) in Iraq in 2007 [55] and in 2011–2012 [54]. In Saudi Arabia, in women of childbearing age sampled from general populations, the pooled T2DM prevalence estimated at 8.0% (95% CI, 5.3–11.3%, *I*^2^, 96.5%) (Table 1). In Saudi Arabia, the weighted T2DM prevalence in women of childbearing age, regardless of source of population and timeline, estimated at 7.2% (95% CI, 4.6–10.2%, *I*^2^, 98.6%) (Table 2). In Oman, the weighted T2DM prevalence in women of childbearing age sampled from general populations estimated at 8.0% (95% CI, 2.9–15.4%, *I*^2^, 95.9%) in 2000. In Qatar, the weighted T2DM was prevalence in women of childbearing age sampled from general populations 10.7% (95% CI, 2.2–24.4%, *I*^2^, 93.7%) between 2007 and 2008. In the UAE, in women of childbearing age sampled from general populations, the pooled T2DM prevalence estimated at 8.0% (95% CI, 4.8–11.9%, *I*^2^, 98.9%) that declined from 9.4% (95% CI, 5.6–14.1%, *I*^2^, 95.1%) between 2000 and 2009 to 6.0% (95% CI, 3.3–6.5%, *I*^2^, 90.5%) between 2010 and 2018 (Table 2).

#### Sub-regional pooled T2DM prevalence

The pooled T2DM prevalence measures estimated at 6.5% (95% CI, 4.3–9.1%, *I*^2^, 96.0%) in North African countries including Iran, 10.7% (95% CI 5.2–17.7%, *I*^2^, 90.7%) in the Fertile Crescent countries, and 7.6% (95% CI, 5.9–9.5%, *I*^*2*^, 98.5%) in the Arabian Peninsula countries (see Additional file 6).

Additional file 7 shows figures presenting the sub-regional-weighted prevalence of T2DM (Fig. 1) in women of childbearing age from 2000 to 2009 and from 2010 to 2018. Additional file 8 shows figures presenting timeline view of the weighted prevalence of T2DM (Fig. 1) by publication year.

#### Meta-bias in T2DM prevalence

The asymmetry in the funnel plot examining the small-study effects on the pooled T2DM prevalence among women of childbearing age indicates evidence for the presence of a small-study effect (Egger’s test *p* < 0.0001). The funnel plot is presented in an additional figure file (see Additional file 3).

#### Predictors of heterogeneity in T2DM prevalence

In the univariate meta-regression models, all variables except study period, T2DM ascertainment criteria, and sample size were associated with T2DM prevalence at *p* value < 0.1. In the adjusted meta-regression model, none of the included variables was significantly associated with T2DM prevalence at *p* value < 0.05. In two studies in infertile women of childbearing age in Egypt, the T2DM prevalence was higher (adjusted odds ratio (aOR), 5.26, 95% CI, 0.87–32.1) compared to women of childbearing age in Saudi Arabia. Overall, compared to women of childbearing age sampled from general populations, T2DM prevalence in non-pregnant women of childbearing age with a history of GDM was 234% higher (aOR, 3.34%, 95% CI, 0.90–12.41) (see Additional file 9).

### Scope of reviewed pre-DM reports

The 24 research reports on pre-DM prevalence yielded 52 pre-DM prevalence studies and were from 10 countries (Iran, Iraq, Jordan, Kuwait, Morocco, Oman, Qatar, Saudi Arabia, UAE, and Yemen); ranging by year between 2002 in Oman [62] and 2018 in Saudi Arabia [81]. Thirteen (25.0%), 11 (21.2%), and 11 (21.2%) of the pre-DM prevalence studies were from Iran, Saudi Arabia, and UAE, respectively. Approximately 87.0% of the pre-DM prevalence studies tested women of childbearing age sampled from general populations. The pre-DM prevalence estimates ranged from 0.0% in various age groups in multiple countries [51, 60, 70] to 40.0% in Iraq in women aged 20–39 years, recruited from the general population [55] (Table 1).

#### Pooled pre-DM prevalence

**Fig. 3:**
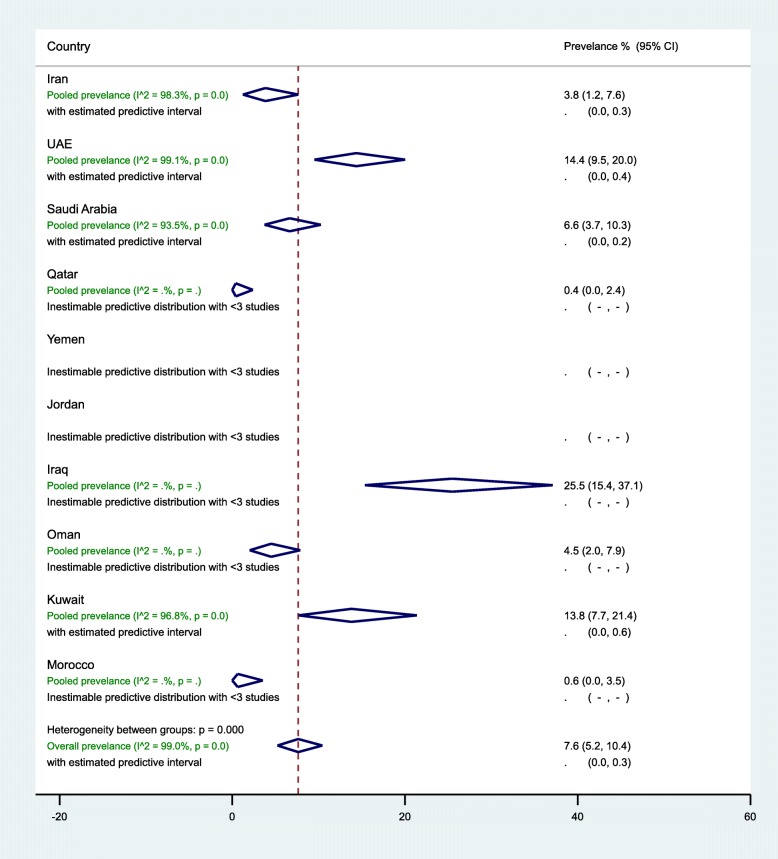
Forest plot of the meta-analyses for the 10 MENA countries’ studies on pre-DM pooled findings of 52 pre-DM prevalence estimates reported in 10 countries in the MENA region. The individual 52 estimates and their 95% confidence interval (CI) omitted to fit the plot. The diamond is centered on the summary effect estimate, and the width indicates the corresponding 95% CI. UAE, United Arab Emirates; pre-DM, pre-diabetes mellitus; MENA, Middle East and Northern Africa

In the 10 countries, the weighted pre-DM prevalence in women of childbearing age was estimated at 7.6% (95% CI, 5.2–10.4%, *I*^2^, 99.0%) (Table 3, Fig. 3). The weighted pre-DM prevalence in studies reported between 2000 and 2009 (4.8%, 95% CI 4.0–7.8%, *I*^2^, 97.1%) was significantly lower (*p* < 0.001) than the weighted prevalence estimated in studies reported between 2010 and 2018 (9.3%, 95%, 4.7–15.2%, *I*^2^, 93.9%) (Table 3). Weighted pre-DM prevalence was 1.70 times higher in women with an age range of 15–19 years (9.0%, 95% CI, 4.9–14.1%, *I*^2^, 99.2%) than women with an age range of 30–49 years (5.3%, 95% CI, 1.8–10.3%, *I*^2^, 99.0%) (see Additional file 5).

**Table 3:** Weighted national prevalence of pre-DM in childbearing age women in MENA countries

Country/population type	No. of studies	Tested sample	pre-DM	pre-DM prevalence					Heterogeneity measures		
				Range (%)	Median (%)	Weighted %	95% CI	Subgroup *p* value	Q (*p* value)^1^	*I*^2^ (%)^2^	95% prediction interval (%)^3^
Iran											
General population^4^	7	4998	222	0.0–7.8	2.0	2.5	0.9–4.7	0.6	80.3 (*p* < 0.001)	92.5	0.0–10.0
Pregnant	4	4832	587	1.7–21.4	4.4	6.6	0.4–19.2		483.1 (*p* < 0.001)	99.4	0.0–90.0
Non-pregnant with a history of GDM	2	173	8	0.9–11.1	6.0	3.4	1.0–6.8		NE	NE	NE
Study period^5^											
2000–2009	12	9893	816	0.0–21.4	3.7	4.1	1.3–8.2	0.1	707.7 (*p* < 0.001)	98.4	0.0–30.0
Overlapping^6^	1	110	1	0.9	NE	NE	NE		NE	NE	NE
Overall^7^	13	10,003	817	0.0–21.4	3.4	3.8	1.2–7.6		717.5 (*p* < 0.001)	98.3	0.0–30.0
Iraq											
General population^4^	3	1370	340	40.0–27.3	15.7	25.5	15.4–37.1	NE	25.5 (*p* < 0.001)	92.2	NE
Study period^5^											
2000–2009	1	40	16	40.0	NE	NE	NE	< 0.001	NE	NE	NE
2010–2018	2	1332	324	15.6–27.4	21.5	24.1	21.8–26.4		35.9 (*p* < 0.001)	NE	NE
Jordan											
General population^4^ (2012–2013)	1	71	10	14.1	NE	NE	NE	NE	NE	NE	NE
Kuwait											
General population^4^ (2000–2009)	4	3256	374	8.2–30.9	10.4	13.8	7.7–21.4	NE	96.8 (*p* < 0.001)	96.8	0.0–60.0
Morocco											
General population^4^ (2000–2009)	2	113	1	0.0–1.4	0.7	0.6	0.0–3.5	NE	NE	NE	NE
Oman											
General population^4^ (2000–2009)	3	2088	82	6.4–6.0	6.0	4.5	2.0–7.9	NE	NE	NE	NE
Qatar											
General population^4^ (2007–2008)	3	471	4	0.0–2.4	0.0	0.4	0.0–2.4	NE	NE	NE	NE
Saudi Arabia											
General population^4^	11	5257	358	0.0–26.0	4.4	6.6	3.7–10.3	NE	154.2 (*p* < 0.001)	93.5	0.0–20.0
Study period^5^											
2000–2009	5	4629	325	1.7–26.0	8.6	9.4	4.5–15.9	0.1	139.4 (*p* < 0.001)	97.1	0.0–40.0
2010–2018	6	628	33	0.0–9.8	3.8	4.4	1.9–7.8		13.0 (*p* < 0.001)	61.5	0.0–20.0
United Arab Emirates											
General population^4^	10	24,723	6071	5.0–35.3	13.9	15.5	10.5–21.2	< 0.001	942.5 (*p* < 0.001)	99.0	0.0–40.0
Non-pregnant with a history of GDM	1	549	30	5.5	NE	NE	NE		NE	NE	NE
Study period^5^											
2000–2009	4	2904	205	5.0–8.7	6.1	6.6	5.1–8.3	< 0.001	8.7 (*p* < 0.001)	65.6	0.0–10.0
2010–2018	3	429	52	9.0–15.3	12.6	12.0	8.9–15.5		NE	NE	NE
Overlapping^6^	4	21,939	5844	20.1–35.3	25.7	16.7	20.5–33.5		296.9 (*p* < 0.001)	99.0	0.0–60.0
Overall^7^	11	25,272	6101	5.0–35.3	12.6	14.4	9.5–20.0		1104.5 (*p* < 0.001)	99.1	0.0–40.0
Yemen											
General population^4^ (before 2010)	1	54	2	3.7	NE	NE	NE	NE	NE	NE	NE
All countries											
Population											
General population^4^	45	42,404	7464	0.0–40.0	6.7	7.9	5.3–11.0	0.6	4478.6 (*p* < 0.001)	99.0	0.0–40.0
Pregnant	4	4832	587	1.7–21.4	4.4	6.6	0.4–19.2		483.1 (*p* < 0.001)	99.4	0.0–90.0
Non-pregnant with a history of GDM	3	722	38	0.9–11.1	5.5	4.7	1.1–10.4		NE	NE	NE
Study period^5^											
2000–2009	35	23,448	1825	0.0–40.0	5.0	4.8	4.0–7.8	< 0.001	1188.4 (*p* < 0.001)	97.1	0.0–20.0
2010–2018	12	2460	419	0.0–27.3	9.4	9.3	4.7–15.2		180.4 (*p* < 0.001)	93.9	0.0–40.0
Overlapping^6^	5	22,050	5845	0.9–35.3	21.9	20.7	15.0–27.1		376.6 (*p* < 0.001)	98.9	0.0–50.0
Ascertainment^9^											
WHO guidelines	19	11,335	837	0.0–15.3	6.7	6.2	4.7–7.9	< 0.001	200.6 (*p* < 0.001)	91.0	0.0–20.0
ADA guidelines	23	33,469	7148	0.0–40.0	11.1	11.3	7.2–16.1		3036.3 (*p* < 0.001)	99.0	0.0–40.0
Carpenter and Coustan	3	2416	70	1.7–8.9	3.9	3.2	1.5–5.4		NE	NE	NE
Medical records	6	738	34	0.0–9.8	3.3	3.7	1.5–6.8		17.9 (*p* < 0.001)	66.5	0.0–20.0
Sample size											
< 100	10	586	44	0.0–15.3	4.1	4.9	1.8–9.2	0.3	32.3 (*p* < 0.001)	72.1	0.0–20.0
≥ 100	42	47,372	8045	0.0–40.0	6.4	8.3	5.6–11.5		5102.9 (*p* < 0.001)	99.2	0.0–40.0
Overall^10^	**52**	47,958	8089	0.0–40.0	6.0	7.6	5.2–10.4		5176.6 (*p* < 0.001)	99.0	0.0–30.0

^1^Q: Cochran’s *Q* statistic is a measure assessing the existence of heterogeneity in estimates of pre-DM prevalence

^2^*I*^2^: a measure assessing the percentage of between-study variation that is due to differences in pre-DM prevalence estimates across studies rather than chance

^3^Prediction interval: estimates the 95% confidence interval in which the true pre-DM prevalence estimate in a new study is expected to fall

^4^General populations could include healthy population, health care workers, migrant workers, or employees

^5^Year range does not cover every single year within that range. In studies with unclear information on when the study was conducted, we subtracted 2 years from the publication year as this was the median of the data collection period and the publication year for the other studies with full information

^6^Study period was before and after 2009

^7^Pooled estimate, regardless of the tested population, sample size, and data collection period, used the most updated criteria when pre-DM was ascertained, based on different criteria in the same population

*NE* not estimable, *CI* confidence interval calculated using the exact binomial method, *pre-DM* pre-diabetes mellitus, *GDM* gestational diabetes, *WHO* World Health Organization, *GCT* glucose challenge test, *OGTT* oral glucose tolerance test, *DM* diabetes mellitus, *T2DM* type 2 diabetes, *ADA* American Diabetes Association, *IDF* International Diabetes Federation, *HIV* human immunodeficiency syndrome, *COPD* chronic obstructive pulmonary disease; *PCOS* polycystic ovary syndrome

In general populations, the highest three weighted pre-DM prevalence estimates were observed in women of childbearing age in Iraq (25.5%, 95% CI, 15.4–37.1%, *I*^*2*^, 92.2%), followed by UAE (15.5%, 95% CI, 10.5–21.2%, *I*^*2*^, 99.0%), and Kuwait (13.8%, 95% CI, 7.7–21.4%, *I*^*2*^, 96.8%) (Table 3). In 13 studies in Iran (7 from the general population), the prevalence of pre-DM ranged from 0.0 to 21.4% with an overall weighted prevalence of 3.8% (95% CI, 1.2–7.6%, *I*^2^*,* 98.3%). The 11 pre-DM studies in Saudi Arabia were in women of childbearing age sampled from the general population, with an overall weighted pre-DM prevalence of 6.6% (95% CI, 3.7–10.3%, *I*^2^, 93.5%) (2000–2009: 9.4% vs. 2010–2018: 4.4%). Regardless of the tested population in UAE, the weighted pre-DM prevalence was 6.6% (95% CI, 5.1–8.3%, *I*^2^, 65.6%) in studies reported between 2000 and 2009, and 12.0% (95% CI, 8.9–15.5%) in studies reported between 2010 and 2018 with an overall pre-DM prevalence of 14.4% (95% CI, 9.5–20.0%, *I*^2^, 99.1%) (Table 3).

#### Sub-regional pooled pre-DM prevalence

The pooled pre-DM prevalence estimated at 3.3% (95% CI, 1.0–6.7%, *I*^2^, 98.1%) in North African countries including Iran, 22.7% (95% CI, 14.2–32.4%, *I*^2^*,* 90.0%) in the Fertile crescent countries, and 8.6% (95% CI, 5.5–12.1%, *I*^*2*^*,* 99.1%) in the Arabian Peninsula countries (see Additional files 10). Additional file 7 shows figures presenting the sub-regional weighted prevalence of pre-DM (Fig. 2) in women of childbearing age from 2000 to 2009 and from 2010 to 2018. Additional file 8 shows figures presenting timeline view of the weighted prevalence of pre-DM (Fig. 2) by publication year.

#### Meta-bias in pre-DM prevalence measures

The asymmetry in the funnel plot examining the small-study effects on the pooled pre-DM prevalence among women of childbearing age indicates evidence for the presence of a small-study effect (Egger’s test *p* < 0.0001). The funnel plot is presented in an additional figure file (Additional file 4).

#### Predictors of heterogeneity in pre-DM prevalence

Country, study period, and pre-DM ascertainment criteria were associated with a difference in the pre-DM prevalence in the univariate meta-regression models at *p* value < 0.1. In the univariate meta-regression models, pre-DM prevalence in women of childbearing age in Iraq was 424% higher compared to such women in Saudi Arabia (OR, 5.24, 95% CI, 1.45–18.94%). This significant association turned insignificant in the multivariable model (aOR, 2.20, 95% CI, 0.52–10.82%). In the multivariable model, compared to Saudi Arabia, pre-DM prevalence in women of childbearing age was 70% lower in Iran (aOR, 0.30, 95% CI, 0.11–0.79%) and 88% lower in Morocco (aOR, 0.12, 95% CI, 0.01–0.91%) (see Additional file 11).

### Quality assessment of the T2DM/pre-DM research reports

Findings of our summarized and research report-specific quality assessments for relevant DM prevalence studies can be found in Additional file 12. Briefly, all the 48 research reports clearly stated their research questions or objectives, clearly specified and defined their study populations, and selected or recruited the study subjects from the same or similar populations. There was a clear gap in the reporting or justifying of the sample size calculation in 79.2% of the research reports. The majority (87.5%) of the research reports tested ≥ 100 women of childbearing age, and they were classified as having high precision.

Overall, the 48 research reports were of reasonable quality with potentially low ROB in an average of 7.2 items (range, 6–9). Four (8.3%) of the 48 reports had potentially low ROB in all the measured nine quality items [66, 82, 83, 86] (see Additional file 12).

## Discussion

We provided, to our knowledge, the first regional study that comprehensively reviewed and estimated the regional, sub-regional, and country-level burden of T2DM and pre-DM in various populations of women of childbearing age in the MENA. Based on the available data from 14 and 10 studies in MENA countries, the present findings document the comparable burden of T2DM (7.5%, 95% CI 6.9–9.0%) and pre-DM (7.6%, 95% CI 5.2–10.4%) in women of childbearing age. The estimated prevalence of T2DM and pre-DM in 14 countries in the MENA is similar to the estimated worldwide crude diabetes prevalence of 8.2% (95% credible interval (CI) 6.6–9.9%) in adult women in 2014 (age-standardized 7.9%, 95% CI 6.4–9.7%) [91]. The T2DM and pre-DM prevalence in women of childbearing age varied across the three sub-regions in the MENA, by population group, time period, DM ascertainment criteria, and sample size. The obvious common prevalence of T2DM and pre-DM in women of childbearing age in the MENA countries reflects the highest prevalence of adult diabetes estimated for the MENA [91]. In this region, the crude diabetes prevalence in adult women increased from 5.0% in 1980 to 9.0% in 2014 [91]. This increase in diabetes prevalence among adult populations in the MENA over time is higher than many other regions including Europe and Central and West Africa [91]. The highest national adult diabetes prevalence estimates documented in the MENA is 5–10 times greater than the lowest national prevalence estimates documented in Western European countries [91].

T2DM is a significant public health problem in both developed and developing countries that can lead to various health complications including increased overall risk of dying prematurely [20]. The common burden of T2DM and pre-DM in women of childbearing age, which is reflected in the high burden of adult diabetes in this region [91], might be mainly driven by the sociodemographic changes in this region. In recent decades, there was an increase in median age, sedentary lifestyle, and physical inactivity in the MENA [92]. These lifestyle changes are linked to an increase in the burden of body overweight and obesity that are shared predisposing factors for pre-DM and T2DM [20]. At the population level, physical inactivity was very common in many MENA countries (Saudi Arabia 67.6% in 2005; Kuwait 62.6% in 2014; Qatar 45.9% in 2012; Egypt 32.1% in 2011–2012; Iraq 47.0% in 2015) [25]. The burden of body overweight and obesity is higher in many low-income and middle-income countries in the MENA than in Europe and Asia Pacific countries [93]. Obesity in women in several Middle Eastern countries was 40–50% [93]. The age-standardized prevalence of obesity was 32.0% in Egypt, 35.5% in Jordan, 30.4% in Iraq, 32.5% in Libya, and 35.4% in Saudi Arabia [94]. In Tunisia, 43.7% and 24.1% of 35–70-year-old females in urban and rural areas, respectively, were obese [95]. In 2016, in almost all of the countries in MENA, the mean BMI for people aged ≥ 18 years was ≥ 25.0 [96].

To curb the burden of DM and its associated complications in women of childbearing age in the MENA countries, our results suggest three main implications for care. First, based on the estimated 5–10% progression rate from pre-DM to T2DM [3, 10], out of the 47,958 tested women of childbearing age for pre-DM (Table 3), we estimate that 2398 to 4796 women are expected to progress to T2DM. This risk of progression to T2DM could be reduced through lifestyle and drug-based interventions as it was reported elsewhere [97–99]. In England, 55–80% of participants with hyperglycemia at baseline had normal glycaemia at 10 year follow-up [3]. The high burden of DM along with pre-DM in women of childbearing age could accelerate maternal complications including GDM leading to increased intergenerational risk of DM. Programs to halt the growing epidemic of DM among different population groups could start by addressing the key risk factors including sedentary lifestyle and increased body weight. Addressing this problem would require social and public policies and efforts to reduce the national and regional burden of increased body weight and obesity through enhancing healthy eating behaviors and physical activity. Second, there is a critical need for strengthened surveillance systems that match the scale and nature of the DM epidemic in women of childbearing age in the MENA. Enhancing early detection and management of high-risk individuals requires accessible and affordable health care systems, outreach campaigns to raise public awareness, and social and medical support to induce and maintain a healthy lifestyle. Adult people at increased risk of T2DM and pre-DM can be predicted based on good screening tools from the Centers for Disease Control and Prevention (CDC) [100] and the American Diabetes Association (T2DM Risk Test) [101]. Early screening and detection will require government-funded prevention programs. Third, controlling the burden of T2DM and pre-DM in MENA countries requires strong and successful partnerships between public health and clinical departments. Physicians have a fundamental role in the care of individual patients to screen, diagnose, and treat both pre-DM and T2DM in clinical settings. In addition, physicians have a fundamental role in working to raise awareness and participating in developing prevention programs and engaging communities. Concerted efforts and partnership between physicians, health departments, and community agencies are needed to strengthen health care services, encouraging and facilitating early screening and detection, and promoting healthy diets and physical activity.

Providing summary estimates and up-to-date mapping gaps-in-evidence of T2DM and pre-DM prevalence in women of childbearing age in different MENA countries provides the opportunities for future public health interventions and research to better characterize the T2DM and pre-DM epidemiology nationally and regionally. Nevertheless, present review findings suggest that the DM burden in women of childbearing age in MENA countries is capturing only the tip of the iceberg. Identifying gaps-in-evidence through systematically reviewing and summarizing the literature has public health research implications. Our review shows that in many countries, the estimation of the burden of T2DM or pre-DM in women of childbearing age in general populations occurred more than a decade ago (Table 1). Additionally, the review shows that there was no data on the burden of T2DM and pre-DM in women of childbearing age in several countries in the MENA region. This lack of evidence on a key public heath outcome requires a strongly resourced research capacity and research funding schemes. There is evidence that federally funded research can impact important health issues that affect a large segment of the population [102].

### Strengths

This robust approach to the literature search and review as well as in retrieving and extracting relevant data from the published literature allowed us to provide summary estimates on the burden of T2DM and pre-DM in women of childbearing age from the 14 and 10 countries in the MENA, respectively. Once the diagnosis was established, regardless of the ascertainment criteria, patients were treated as having diabetes or pre-diabetes. Thus, generating pooled estimates, regardless of the DM ascertainment criteria, stratified according to various population groups, provided more insights into the actual burden of T2DM and pre-DM in various populations of women of childbearing age. The meta-regression analysis identified sources of variations in T2DM and pre-DM prevalence and sources of between-study heterogeneity in prevalence estimates. (Additional files 9 and 11 show these in more detail). The country-stratified and population-stratified T2DM and pre-DM prevalence reports revealed gaps in evidence that can help strengthen research and DM control programs in the most affected countries and populations. The use of probability sampling was very common in the studies included, which may provide broader insights on the representation of our findings to the general or specific group of women of childbearing age at the national, but not at the regional, level.

### Limitations

There are important but unavoidable limitations when interpreting the results of our review. Despite the estimated DM prevalence, the actual DM burden could have been underestimated, at country, sub-regional, or regional level, due to several reasons. The inaccessibility of data on pre-DM or T2DM in women of childbearing age from several countries in the MENA may not necessarily mean an actual lack of data. To meet the aim of our review of estimating the burden of pre-DM and T2DM in women of childbearing age, in several published studies reviewed, women of childbearing age were found to have been combined with those of other age groups or with men. The presented overall pooled estimates, regardless of the tested population group, should not be interpreted as the total burden of the outcome at the population level. Utilizing data on T2DM and pre-DM from only 14 and 10 countries may limit the findings from being generalizable to the entire MENA region. Although we followed a thorough and well-defined search strategy, there is a potential of publication bias as shown in funnel plots (Additional files 3 and 4). The estimated T2DM and pre-DM prevalence suggest that only the tip of the iceberg was captured. The presented estimates may not be representative of the true prevalence for each population. This underestimation may be particularly true in low-resource settings where necessary resources and capacity in investigating pre-DM at the community level are lacking. The wide array of blood glucose cut-off points and criteria used for T2DM and pre-DM ascertainment also suggests that overestimation and underestimation bias cannot be excluded. Unless estimated from individual population-based studies only, the presented weighted pooled estimates at the country, sub-regional, or regional level should not be interpreted as the burden of the measured outcomes at the population level. Also, the presented pooled estimates according to the two time periods, from 2000 to 2009 and from 2010 to 2018, should not be interpreted as an over-time change in the burden of the measured outcomes. While our meta-analyses revealed substantial heterogeneity across studies, the meta-regression analyses identified the potential sources of between-study heterogeneity within the framework of the present study and the level of detail that can be used in describing these sources (Tables 1 and 2). Thus, much of the variability in T2DM and pre-DM prevalence across studies might remain unexplained.

Despite these potential limitations, our study provided a characterization of the scale of T2DM and pre-DM among women of childbearing age in several MENA countries based on the best available evidence. Data presented in this review can be used to (a) understand the burden of T2DM and pre-DM among a vital population group and to identify at high-risk populations within this specific population group; (b) guide the planning, implementation, and evaluation of programs to prevent and control DM; (c) implement immediate public health actions to prioritize the allocation of public health resources; and (d) formulate research hypotheses and provide a basis for epidemiologic studies. Future research opportunities should prioritize large country-level and multicenter comparable studies, to determine the prevalence of T2DM and pre-DM in various population groups of women of childbearing age. A definitive characterization of the burden of DM in women of childbearing age at the regional and sub-regional level would require comparable and empirical studies using standardized methodology and comparable DM ascertainment assays.

## Conclusions

In conclusion, women of childbearing age in the MENA region bear an appreciable burden of T2DM and pre-DM. The estimated burden of T2DM and pre-DM was higher in the Arabian Peninsula and Fertile Crescent countries compared to the rest of the MENA countries identified with prevalence estimates in this review. Although both T2DM (7.5%) and pre-DM (7.6%) had similar overall estimated prevalence, there is need for a more focused attention on early detection and control by public health authorities to avoid DM-associated pre-gestational, gestational, and post-gestational complications. Country-level early DM detection and control programs should consider the key risk factors of DM, mainly the growing burden of body overweight and obesity. Furthermore, facilitating high-quality research and surveillance programs in countries with limited data on DM prevalence and reporting of DM prevalence estimates in women of childbearing age warrant focus.

## Supplementary information

**Additional file 1.** PRISMA checklist.

**Additional file 2.** Search strategies for the six databases, from January 1, 2000 to July 12, 2018.

**Additional file 3 **Funnel plots examining small-study effects on the pooled T2DM prevalence among women of childbearing age. Egger’s test *p*<0.0001.

**Additional file 4 **Funnel plots examining small-study effects on the pooled pre-DM prevalence among women of childbearing age. Egger’s test *p*<0.0001.

**Additional file 5.** Weighted prevalence of T2DM and pre-DM in childbearing age women in MENA countries according to age group.

**Additional file 6.** Sub-regional weighted prevalence of T2DM in women of childbearing age according to the tested population, data collection period, T2DM ascertainment, sample size, and overall, in 14 MENA countries.

**Additional file 7.** Sub-regional weighted prevalence of T2DM (Figure 1) and pre-DM (Figure 2) in women of childbearing age from 2000 to 2009 and from 2010 to 2018. Square represents the estimated prevalence and lines around the square represent the upper and lower limit of the 95% confidence interval of the prevalence.

**Additional file 8.** Timeline view of the weighted prevalence of T2DM (Figure 1) and pre-DM (Figure 2) in women of childbearing age, by publication year.

**Additional file 9.** Univariate and multivariable meta-regression analyses to identify sources of heterogeneity in studies reporting on T2DM prevalence in women of childbearing age by the different measured characteristics.

**Additional file 10.** Sub-regional weighted prevalence of pre-DM in childbearing age women according to the tested population, data collection period, Pre-DM ascertainment, sample size, and overall, in the four sub regions of the 10 MENA countries.

**Additional file 11.** Univariate and multivariable meta-regression analyses to identify sources of heterogeneity in studies reporting on pre-DM prevalence in women of childbearing age by the different measured characteristics.

**Additional file 12.** Quality assessment of the 48 research reports included in the analysis.

## Data Availability

The datasets used and/or analyzed during the current study and its supplementary information files are available from the corresponding author on reasonable request.

## Abbreviations

ADA: American DM association
aOR: Adjusted odds ratio
CI: Confidence interval
DM: Diabetes mellitus
GDM: Gestational diabetes mellitus
IDF: International Diabetes Mellitus Association
MENA: Middle East and North Africa
MeSH: Medical Subject Headings
NIH: National Heart, Lung, and Blood Institute
PECO: Participants, exposure, comparator, and outcome
Pre-DM: Pre-diabetes mellitus
PRISMA: Preferred Reporting Items for Systematic Review and Meta-Analysis
ROB: Risk of bias
T2DM: Type 2 diabetes
UAE: United Arab Emirates
WHO: World Health Organization
