# Supplementary material for: Type 2 diabetes and pre-diabetes mellitus: a systematic review and meta-analysis of prevalence studies in women of childbearing age in the Middle East and North Africa, 2000–2018
Source: Syst Rev. 2019 Nov 8;8:268. doi: 10.1186/s13643-019-1187-1 (PMC6839168; doi:10.1186/s13643-019-1187-1)
Supplement: Supplementary file 2 — Additional file 2. Search strategies for the six databases, from January 1, 2000 to July 12, 2018. [file 13643_2019_1187_MOESM2_ESM.docx]

**Additional file 2. Search strategies for the six databases, from January 1, 2000 to July 12, 2018**

**MEDLINE-PubMed**

((("Diabetes Mellitus”[Mesh] OR "Diabetes Mellitus, Type 2"[Mesh] OR “diabetes type 2”[Mesh] OR “T2DM”[Mesh] OR “diabetes Type II”[Mesh] OR diabetes”[Mesh] OR "glucose intolerance"[Mesh] OR "insulin resistance"[Mesh] OR "Hyperglycemia"[Mesh] OR "Hypoglycemia"[Mesh] OR OR "Diabetes Mellitus”[Text] OR "Diabetes Mellitus, Type 2"[Text] OR “diabetes type 2”[Text] OR “T2DM”[Text] OR “diabetes Type II”[Text] OR diabetes”[Text] OR "glucose intolerance"[Text] OR "insulin resistance"[Text] OR "Hyperglycemia"[Text] OR "Hypoglycemia"[Text]) AND ("Adolescent"[Mesh] OR "Young Adult"[Mesh] OR "Adult"[Mesh] OR "Middle Aged"[Mesh] OR “teenage”[Mesh] OR “adolescent”[Mesh] OR “young adult”[Mesh] OR “adult”[Mesh] OR “Middle Age”[Mesh] OR "Adolescent"[Text] OR "Young Adult"[Text] OR "Adult"[Text] OR "Middle Aged"[Text] OR “teenage”[Text] OR “adolescent”[Text] OR “young adult”[Text] OR “adult”[Text] OR “Middle Age”[Text]) AND ("Middle East"[Mesh] OR "Islam"[Mesh] OR "Arabs"[Mesh] OR "Arab World"[Mesh] OR "Africa, Northern"[Mesh] OR "Sudan"[Mesh] OR "Somalia"[Mesh] OR "Djibouti"[Mesh] OR "Middle East"[Text] OR "Middle-East"[Text] OR "North Africa"[Text] OR "North-Africa"[Text] OR "EMRO"[Text] OR "Eastern Mediterranean"[Text] OR “Arab”[Text] OR “Arabs”[Text] OR “Arab World”[Text] OR "Islam"[Text] OR "Afghanistan"[Text] OR "Algeria"[Text] OR "Bahrain"[Text] OR "Djibouti"[Text] OR "Egypt"[Text] OR "Jordan"[Text] OR "Kuwait"[Text] OR "Lebanon"[Text] OR "Libya"[Text] OR "Iran"[Text] OR "Iraq"[Text] OR "Morocco"[Text] OR "Oman"[Text] OR "Qatar"[Text] OR "Saudi Arabia"[Text] OR "Somalia"[Text] OR "Sudan"[Text] OR "Syria"[Text] OR "Tunisia"[Text] OR "United Arab Emirates"[Text] OR "Dubai"[Text] OR "Abu Dhabi"[Text] OR "Abu-Dhabi"[Text] OR “Sharjah”[Text] OR "West Bank"[Text] OR "Ghaza"[Text] OR "Palestine"[Text] OR "Yemen"[Text])))

Search filter: Human studies

**Embase**

(((‘Diabetes Mellitus, Type 2’/exp OR ‘Diabetes Mellitus, Type II'/exp OR ‘diabetes type 2’/exp OR ‘T2DM’/exp OR ‘diabetes Type II’/exp OR ‘diabetes’/exp OR ‘hyperglycemia’/exp OR ‘insulin resistance’/exp OR ‘hypoglycemia’/exp OR ‘oral glucose tolerance’/exp)) AND ((‘Adolescent’/exp OR ‘Young Adult’/exp OR ‘Adult’/exp OR ‘Middle Aged’/exp OR teenage* OR adolescen*)) AND ((‘Middle East’/exp OR ‘North Africa’/exp OR ‘Arab’/exp OR ‘Afghanistan’/exp OR ‘Djibouti’/exp OR ‘Somalia’/exp OR ‘Sudan’/exp OR ‘EMRO’/exp OR ‘Eastern Mediterranean’/exp OR ‘Arabs’/exp OR ‘Arab World’/exp OR ‘Islam’/exp OR ‘Algeria’/exp OR ‘Bahrain’/exp OR ‘Egypt’/exp OR ‘Jordan’/exp OR ‘Kuwait’/exp OR ‘Lebanon’/exp OR ‘Libya’/exp OR ‘Iran’/exp OR ‘Iraq’/exp OR ‘Morocco’/exp OR ‘Oman’/exp OR ‘Qatar’/exp OR ‘Saudi Arabia’/exp OR ‘Syria’/exp OR ‘Tunisia’/exp OR ‘United Arab Emirates’/exp OR ‘Dubai’/exp OR ‘Abu Dhabi’/exp OR ‘Sharjah’/exp OR ‘West Bank’/exp OR ‘Ghaza’/exp OR ‘Palestine’/exp OR ‘Yemen’/exp)))

Search filters

Publication type (Article; article in press; Conference Abstract; Conference Paper; Conference Review

Language: English, Arabic.

Gender: Male and Female

**WEB OF SCIENCE**

TS= (Diabete* OR T2DM OR Hyperglycemia OR "insulin resistance" OR Hypoglycemia OR "glucose intolerance") AND TS= (Adolescen* OR "Young Adult" OR Adult OR "Middle Aged" OR teenage*) AND TS= ("Middle East" OR "North Africa" OR Arab OR Afghanistan OR Djibouti OR Somalia OR Sudan OR EMRO OR "Eastern Mediterranean" OR Arabs OR "Arab World" OR Islam OR Algeria OR Bahrain OR Egypt OR Jordan OR Kuwait OR Lebanon OR Libya OR Iran OR Iraq OR Morocco OR Oman OR Qatar OR "Saudi Arabia" OR Syria OR Tunisia OR "United Arab Emirates" OR Dubai OR "Abu Dhabi" OR Sharjah OR "West Bank" OR Ghaza OR Palestine OR Yemen)

Search filters

LANGUAGE: (English OR Arabic)

DOCUMENT TYPES: (Article OR Abstract of Published Item OR Meeting Abstract OR Meeting Summary OR Proceedings Paper)

Timespan= Jan 1, 2000-July 12, 2018

**SCOPUS**

((TITLE-ABS-KEY(diabete! OR t2dm OR hyperglycemia OR "insulin resistance" OR hypoglycemia OR "glucose intolerance") AND TITLE-ABS-KEY(adolescen! OR “Young Adult” OR adult OR “Middle Aged” OR teenage!) AND TITLE-ABS-KEY("Middle East" OR "North Africa" OR Arab OR Afghanistan OR Djibouti OR Somalia OR Sudan OR Bahrain OR Egypt OR Jordan OR Kuwait OR Lebanon OR Libya OR Iran OR Iraq OR Morocco OR Oman OR Qatar OR Saudi Arabia OR Somalia OR Sudan OR Syria OR Tunisia OR United Arab Emirates OR Dubai OR Abu Dhabi OR Sharjah OR West Bank OR Ghaza OR Palestine OR Yemen))

Search filters

Search Field: TITLE-ABS-KEY

LIMIT-TO DOCTYPE: Articles or Conference Paper

LIMIT-TO LANGUAGE: English or Arabic

**COCHRANE LIBRARY**

(Diabete* or T2DM or *glycemia or insulin resistan* or glucose intoleran*) AND (Adolescen* or "Young Adult" or Adult or "Middle Aged" or teenage*) AND ("Middle East" or "North Africa" or Arab or Afghanistan or Djibouti or Somalia or Sudan or EMRO or "Eastern Mediterranean" or Arabs or "Arab World" or Islam or Algeria or Bahrain or Egypt or Jordan or Kuwait or Lebanon or Libya or Iran or Iraq or Morocco or Oman or Qatar or "Saudi Arabia" or Syria or Tunisia or "United Arab Emirates" or Dubai or "Abu Dhabi" or Sharjah or "West Bank" or Ghaza or Palestine or Yemen)

Search filters

Search Field: All Text

**ACADEMIC SEARCH COMPLETE**

((Diabete* OR T2DM OR Hyperglycemia OR "insulin resistance" OR Hypoglycemia OR "glucose intolerance") AND (Adolescen* OR "Young Adult" OR Adult OR "Middle Aged" OR teenage*) AND ("Middle East" OR "North Africa" OR Arab OR Afghanistan OR Djibouti OR Pakistan OR EMRO OR "Eastern Mediterranean" OR Arabs OR "Arab World" OR Islam OR Algeria OR Bahrain OR Egypt OR Jordan OR Kuwait OR Lebanon OR Libya OR Iran OR Iraq OR Morocco OR Oman OR Qatar OR "Saudi Arabia" OR Syria OR Tunisia OR "United Arab Emirates" OR Dubai OR "Abu Dhabi" OR Sharjah OR "West Bank" OR Ghaza OR Palestine OR Yemen))

Search filters

Limiters: References Available; Scholarly (Peer Reviewed) Journals.

Document Type: Abstract, Article, Bibliography, Proceeding.

Search modes: Boolean/Phrase

Select a Field: All Text
