# Supplementary material for: Type 2 diabetes and pre-diabetes mellitus: a systematic review and meta-analysis of prevalence studies in women of childbearing age in the Middle East and North Africa, 2000–2018
Source: Syst Rev. 2019 Nov 8;8:268. doi: 10.1186/s13643-019-1187-1 (PMC6839168; doi:10.1186/s13643-019-1187-1)
Supplement: Supplementary file 4 — Additional file 4 Funnel plots examining small-study effects on the pooled pre-DM prevalence among women of childbearing age. Egger’s test p<0.0001. [file 13643_2019_1187_MOESM4_ESM.docx]

**Additional file 4-** Funnel plots examining small-study effects on the pooled pre-DM prevalence among women of childbearing age; Egger’s test p<0.0001

Pre-DM: pre diabetes mellitus
