# Supplementary material for: Type 2 diabetes and pre-diabetes mellitus: a systematic review and meta-analysis of prevalence studies in women of childbearing age in the Middle East and North Africa, 2000–2018
Source: Syst Rev. 2019 Nov 8;8:268. doi: 10.1186/s13643-019-1187-1 (PMC6839168; doi:10.1186/s13643-019-1187-1)
Supplement: Supplementary file 5 — Additional file 5. Weighted prevalence of T2DM and pre-DM in childbearing age women in MENA countries according to age group. [file 13643_2019_1187_MOESM5_ESM.docx]

**Additional file 5.** Weighted prevalence of T2DM and pre-DM in childbearing age women in MENA countries according to age group

| **Age group** | **T2DM** | | | | | **pre-DM** | | | | |
| --- | --- | --- | --- | --- | --- | --- | --- | --- | --- | --- |
|  | No. of studies | Weighted prev.  (95% CI) | Heterogeneity measures | | | No. of studies | Weighted prev.  (95% CI) | Heterogeneity measures | | |
|  |  |  | Q (*p*−value)^1^ | *I^2^* (%)^2^ | 95% prediction interval (%)^3^ |  |  | Q  (*p*−value)^1^ | *I^2^* (%)^2^ | 95% prediction interval (%)^3^ |
| 15-29 years | 28 | 2.5  (1.8–3.2) | 159.0 (p<0.001) | 83.6 | 0.0–10.0 | 17 | 5.3  (1.8–10.3) | 1574.4 (p<0.001) | 99.0 | 0.0–40.0 |
| 30-49 years | 49 | 10.9  (8.8–13.3%) | 2260.4 (p<0.001) | 97.9 | 0.0–30.0 | 24 | 9.0  (4.9–14.1) | 2984.6 (p<0.001) | 99.2 | 0.0–40.0 |
| Not specified/overlapping | 25 | 7.9%  (5.4–10.9) | 971.4 (p<0.001) | 97.4 | 0.0–30.0 | 11 | 8.5  (4.4–13.5) | 387.1 (p<0.001) | 97.4 | 0.0–30.0 |

^1^ Q: Cochran’s Q statistic is a measure assessing the existence of heterogeneity in estimates of pre-DM prevalence.

^2^ *I*^2^: a measure assessing the percentage of between−study variation that is due to differences in pre-DM prevalence estimates across studies rather than chance.

^3^ Prediction interval: estimates the 95% confidence interval in which the true pre-DM prevalence estimate in a new study is expected to fall.

DM: diabetes mellitus, T2DM: type 2 diabetes mellitus
