# Supplementary material for: Type 2 diabetes and pre-diabetes mellitus: a systematic review and meta-analysis of prevalence studies in women of childbearing age in the Middle East and North Africa, 2000–2018
Source: Syst Rev. 2019 Nov 8;8:268. doi: 10.1186/s13643-019-1187-1 (PMC6839168; doi:10.1186/s13643-019-1187-1)
Supplement: Supplementary file 7 — Additional file 7. Sub-regional weighted prevalence of T2DM (Figure 1) and pre-DM (Figure 2) in women of childbearing age from 2000 to 2009 and from 2010 to 2018. Square represents the estimated prevalence and lines around the square represent the upper and lower limit of the 95% confidence interval of the prevalence. [file 13643_2019_1187_MOESM7_ESM.docx]

**Additional file 7**.


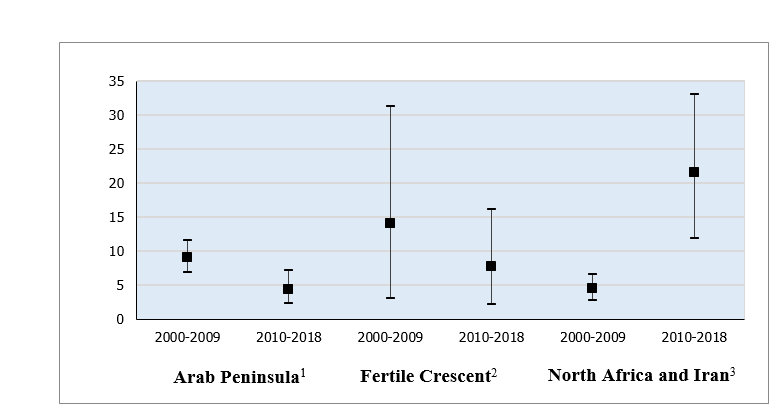


**Figure 1.** Sub-regional weighted prevalence of T2DM in women of childbearing age from 2000 to 2009 and from 2010 to 2018. Square represents the estimated prevalence and lines around the square represent the upper and lower limit of the 95% confidence interval of the prevalence.

^*^ Prevalence of T2DM does not necessarily include prevalence estimates from every single country in every single year in each sub-region within each time period. Thus, the observed difference in prevalence estimate between the two time periods should not be interpreted as an over time change of the sub-regional burden of T2DM in childbearing age women.

^1^ There was no prevalence estimates from Bahrain in both time periods

^2^ There was no prevalence estimates from Palestine in both time periods


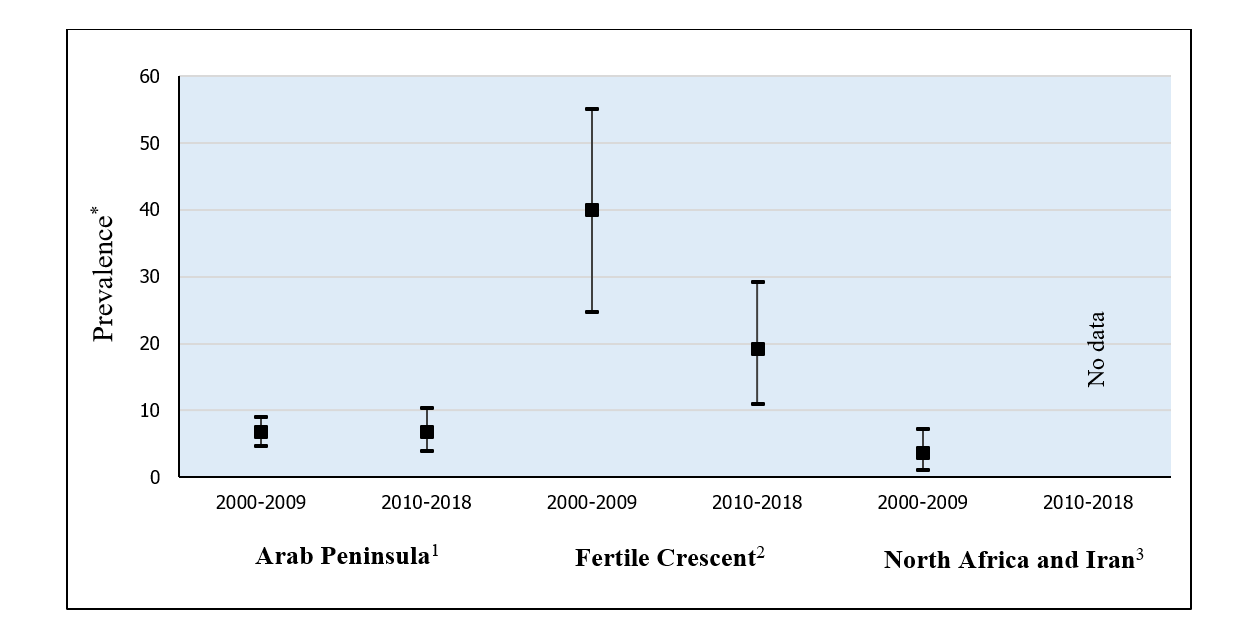
^3^ There was no prevalence estimates from Libya in both time periods

**Figure 2.** Sub-regional weighted prevalence of pre-DM in women of childbearing age from 2000 to 2009 and from 2010 to 2018. Square represents the estimated prevalence estimate and lines around the square represent the upper and lower limit of the 95% confidence interval of the prevalence.

^*^ Prevalence of pre-DM does not necessarily include prevalence estimates from every single country in every single year in each sub-region within each time period. Thus, the observed difference in prevalence estimate between the two time periods should not be interpreted as an over time change of the sub-regional burden of T2DM in childbearing age women. For instance, the observed prevalence of 40% in the Fertile Crescent sub-region represents only one prevalence estimate reported in Iraq in 2007.

^1^ There was no prevalence estimates from Bahrain in both time periods

^2^ There was no prevalence estimates from Palestine in both time periods

^3^ There was no prevalence estimates from Libya in both time periods
