# Supplementary material for: Type 2 diabetes and pre-diabetes mellitus: a systematic review and meta-analysis of prevalence studies in women of childbearing age in the Middle East and North Africa, 2000–2018
Source: Syst Rev. 2019 Nov 8;8:268. doi: 10.1186/s13643-019-1187-1 (PMC6839168; doi:10.1186/s13643-019-1187-1)
Supplement: Supplementary file 8 — Additional file 8. Timeline view of the weighted prevalence of T2DM (Figure 1) and pre-DM (Figure 2) in women of childbearing age, by publication year. [file 13643_2019_1187_MOESM8_ESM.docx]

**Additional file 8**.

Figure 1. Timeline view of the prevalence of T2DM (by publication year) with data points weighted by the sample size of the tested population

No data available for the years 2002 and 2003. Only one data point was available for the years 2000 and 2004

Figure 2. Timeline view of the prevalence of pre-DM (by publication year) with data points weighted by the sample size of the tested population

No data available for the years 2000, 2001, 2006, and 2017. Only one data point was available for the years 2000 and 2004
