# Supplementary material for: Type 2 diabetes and pre-diabetes mellitus: a systematic review and meta-analysis of prevalence studies in women of childbearing age in the Middle East and North Africa, 2000–2018
Source: Syst Rev. 2019 Nov 8;8:268. doi: 10.1186/s13643-019-1187-1 (PMC6839168; doi:10.1186/s13643-019-1187-1)
Supplement: Supplementary file 9 — Additional file 9. Univariate and multivariable meta-regression analyses to identify sources of heterogeneity in studies reporting on T2DM prevalence in women of childbearing age by the different measured characteristics. [file 13643_2019_1187_MOESM9_ESM.docx]

# **Additional file 9**. Univariate and multivariable meta-regression analyses to identify sources of heterogeneity in studies reporting on T2DM prevalence in women of childbearing by the different measured characteristics

|  | **No. of studies** | **Univariate analyses** | | **Multivariable analyses** | |
| --- | --- | --- | --- | --- | --- |
|  |  | OR (95% CI) | *P*–value^†^ | aOR (95% CI) | *P*–value^‡^ |
| **Country** |  |  |  |  |  |
| Algeria | 1 | 0.34 (0.03–4.07) | 0.390 | 0.39 (0.03–4.75) | 0.454 |
| Egypt | 2 | 4.60 (0.77–27.4) | 0.092 | 5.26 (0.87–32.1) | 0.071 |
| Iran | 15 | 0.73 (0.34–1.57) | 0.427 | 0.65 (0.29–1.49) | 0.310 |
| Iraq | 3 | 2.57 (0.58–11.24) | 0.207 | 2.14 (0.44–10.27) | 0.339 |
| Jordan | 1 | 0.42 (0.03–5.00) | 0.485 | 0.27 (0.02–3.64) | 0.320 |
| Kuwait | 4 | 1.03 (0.23–4.51) | 0.967 | 0.67 (0.13–3.47) | 0.629 |
| Lebanon | 2 | 1.11 (0.19–6.58) | 0.911 | 1.26 (0.21–7.67) | 0.799 |
| Morocco | 2 | 0.42 (0.03–5.00) | 0.485 | 0.35 (0.03–4.44) | 0.411 |
| Oman | 2 | 1.39 (0.38–5.09) | 0.610 | 1.04 (0.26–4.21) | 0.952 |
| Qatar | 4 | 1.50 (0.34–6.55) | 0.589 | 0.97 (0.19–5.04) | 0.972 |
| Saudi Arabia^§^ | 38 | 1.00 |  | 1.00 |  |
| Tunisia | 3 | 1.27 (0.30–5.59) | 0.741 | 0.83 (0.16–4.31) | 0.823 |
| United Arab Emirates | 23 | 0.98 (0.51–1.90) | 0.962 | 0.77 (0.36–1.65) | 0.502 |
| Yemen | 1 | 1.46 (0.12–17.60) | 0.761 | 0.95 (0.07–12.8) | 0.969 |
| **Population** |  |  |  |  |  |
| General population^5^ | 81 | 1.00 |  | 1.00 |  |
| Pregnant | 12 | 0.48 (0.25–0.90) | 0.025 | 0.55 (0.27–1.17) | 0.117 |
| Non-pregnant with a history of GDM | 3 | 2.57 (0.77–8.62) | 0.124 | 3.34 (0.90–12.41) | 0.070 |
| Patients^6^ | 4 | 0.61 (0.18–2.04) | 0.421 | 0.58 (0.16–2.11) | 0.407 |
| Infertile | 2 | 4.31 (0.99–18.8) | 0.052 |  |  |
| **Study period**^7^ |  |  |  |  |  |
| 2000–2009 | 71 | 1.00 |  | 1.00 |  |
| 2010–2018 | 26 | 0.87 (0.52–1.48) | 0.519 | – |  |
| Overlapping^8^ | 5 | 1.17 (0.43–3.20) | 0.756 | – |  |
| **Ascertainment**^9^ |  |  |  |  |  |
| Medical records/anti-DM medications/self-reported | 27 | 1.00 |  | 1.00 |  |
| WHO guidelines | 34 | 1.33 (0.76–2.32) | 0.313 | – |  |
| ADA guidelines | 1 | 0.99 (0.58–1.66) | 0.963 | – |  |
| IDF guidelines | 40 | 1.63 (0.18–14.72) | 0.657 | – |  |
| **Sample size** |  |  |  |  |  |
| <100 | 16 | 1.00 |  | 1.00 |  |
| ≥100 | 84 | 0.82 (0.43–1.57) | 0.560 | – |  |

^†^ Statistically significant at p-values <0.1

^‡^ Statistically significant at p-values <0.05

^§^ Reference country

^¶^ Year range in square brackets does not cover every single year within that range.

OR: odds ratio; aOR: adjusted odds ratio; CI, confidence interval calculated using the “exact” method.

CI, confidence interval calculated using the “exact” method.

T2DM: type 2 diabetes mellitus; GDM: gestational diabetes; WHO: World Health Organization; ADA: American Diabetes Association; IDF: International Diabetes Federation
