# Supplementary material for: Type 2 diabetes and pre-diabetes mellitus: a systematic review and meta-analysis of prevalence studies in women of childbearing age in the Middle East and North Africa, 2000–2018
Source: Syst Rev. 2019 Nov 8;8:268. doi: 10.1186/s13643-019-1187-1 (PMC6839168; doi:10.1186/s13643-019-1187-1)
Supplement: Supplementary file 10 — Additional file 10. Sub-regional weighted prevalence of pre-DM in childbearing age women according to the tested population, data collection period, Pre-DM ascertainment, sample size, and overall, in the four sub regions of the 10 MENA countries. [file 13643_2019_1187_MOESM10_ESM.docx]

# **Additional file 10.** Sub-regional weighted prevalence of pre-DM in childbearing age women according to the tested population, data collection period, Pre-DM ascertainment, sample size, and overall, in the four sub regions of the 10 MENA countries

|  | **North Africa and Iran**^1^ | | | | | **Fertile crescent**^2^ | | | | | **Arab peninsula**^3^ | | | | |
| --- | --- | --- | --- | --- | --- | --- | --- | --- | --- | --- | --- | --- | --- | --- | --- |
|  | No. | Range (%) | Prev. % | 95% CI | *I*^2^ (%)^4^ | No. | Range (%) | Prev. % | 95% CI | *I*^2^ (%)^4^ | No. | Range (%) | Prev.% | 95% CI | *I*^2^ (%)^4^ |
| **Population** |  |  |  |  |  |  |  |  |  |  |  |  |  |  |  |
| General population^5^ | 9 | 0.0–7.8 | 2.1 | 0.8–4.0 | 90.5 | 4 | 14.1–40.0 | 22.7 | 14.2–32.4 | 90.0 | 32 | 0.0–35.3 | 8.7 | 5.6–12.3 | 99.1 |
| Pregnant | 4 | 1.7–21.4 | 6.6 | 0.4–19.2 | 99.4 | ND | – | – | – | – | ND | – | – | – | – |
| Non-pregnant with a history of GDM | 2 | 0.9–11.1 | 3.4 | 1.0–6.8 | NE | ND | – | – | – | – | 1 | 5.5 | – | – | – |
| **Study period**^6^ |  |  |  |  |  |  |  |  |  |  |  |  |  |  |  |
| 2000–2009 | 14 | 0.0–21.4 | 3.6 | 1.1–7.2 | 98.2 | 1 | 40.0 | NE | NE | NE | 20 | 0.0–30.9 | 6.7 | 4.7–9.0 | 95.7 |
| 2010–2018 | ND | – | – | – | – | 3 | 14.1–27.4 | 19.2 | 10.9–29.2 | NE | 9 | 0.0–15.3 | 6.8 | 3.9–10.4 | 75.1 |
| Overlapping^7^ | 1 | 0.9 | NE | NE | NE | ND | – | – | – | – | 4 | 20.1–35.3 | 26.7 | 20.5–33.5 | 99.1 |
| **Ascertainment**^8^ |  |  |  |  |  |  |  |  |  |  |  |  |  |  |  |
| WHO guidelines | ND | – | – | – | – | 1 | 14.1 | – | – | – | 18 | 0.0–15.3 | 6.0 | 4.5–7.7 | 91.3 |
| ADA guidelines | 11 | 0.0–21.4 | 3.6 | 0.7–8.2 | 98.3 | 3 | 15.7–40.4 | 25.5 | 15.4–37.1 | NE | 9 | 1.7–35.3 | 19.0 | 12.4–26.7 | 99.4 |
| Carpenter and Coustan | 3 | 1.7–4.9 | 3.2 | 1.5–5.4 |  |  |  |  |  |  |  |  |  |  |  |
| Medical records | 1 | 0.9 | NE | NE | NE | ND | – | – | – | – | 6 | 0.0–9.8 | 4.4 | 1.9–7.8 | 61.5 |
| **Sample size** |  |  |  |  |  |  |  |  |  |  |  |  |  |  |  |
| <100 | 4 | 0.0–11.1 | 2.0 | 0.0–8.2 | 69.5 | 1 | 14.1 | – | – | – | 5 | 0.0–15.3 | 6.0 | 1.8–12.0 | 69.1 |
| ≥100 | 11 | 0.4–21.4 | 3.8 | 1.2–7.8 | 98.6 | 3 | 15.7–40.0 | 25.5 | 15.4–37.1 | NE | 28 | 0.0–44.9 | 9.1 | 5.8–130 | 99.2 |
| **Overall**^9^ | **15** | 0.0–21.4 | **3.3** | **1.0–6.7** | **98.1** | **4** | 14.1–40.0 | **22.7** | **14.2–32.4** | **90.0** | **33** | **0.0–35.3** | **8.6** | **5.5–12.1** | **99.1** |

^1^ North Africa and Iran: Morocco, Algeria, Tunisia, Libya, Egypt, and Iran

^2^ Fertile crescent: Jordan, Syria, Palestine, Lebanon, and Iraq

^3^ Arab peninsula: UAE, Qatar, Saudi Arabia, Kuwait, Yemen, Oman, Bahrain

^4^ *I*^2^: a measure assessing the percentage of between−study variation that is due to differences in *Salmonella* prevalence estimates across studies rather than chance.

^5^ General populations could include healthy population, health care workers, migrant workers, or employees.

^6^ Patients include those on kidney dialysis, or with arthritis, organ transplant, cancer, HIV, COPD, PCOS, or schizophrenia.

^7^ Year range does not cover every single year within that range. In studies with unclear information on when the study was conducted, we subtracted two years from the publication year as this was the median for the other studies with full information.

^8^ Study period was before and after 2009.

^9^ Regardless of the year of the guidelines for the most updated criteria when pre-DM was ascertained, based on different criteria in the same population

^10^ Pooled estimate regardless of the tested population, sample size, and data collection period, using the most updated criteria when pre-DM ascertained using different criteria in the same population

ND: No data

NE: not estimable (only one study, or <2 or <3 studies)

CI: confidence interval calculated using the exact binomial method

Pre-DM: pre diabetes mellitus; GDM: gestational diabetes; WHO: World Health Organization; ADA: American Diabetes Association; ADA: American Diabetes Association; IDF: International Diabetes Federation; HIV: human immunodeficiency syndrome; COPD: chronic obstructive pulmonary disease; PCOS: polycystic ovary syndrome; UAE: United Arab Emirate; MENA: Middle East and Northern Africa
