# Supplementary material for: Type 2 diabetes and pre-diabetes mellitus: a systematic review and meta-analysis of prevalence studies in women of childbearing age in the Middle East and North Africa, 2000–2018
Source: Syst Rev. 2019 Nov 8;8:268. doi: 10.1186/s13643-019-1187-1 (PMC6839168; doi:10.1186/s13643-019-1187-1)
Supplement: Supplementary file 11 — Additional file 11. Univariate and multivariable meta-regression analyses to identify sources of heterogeneity in studies reporting on pre-DM prevalence in women of childbearing age by the different measured characteristics. [file 13643_2019_1187_MOESM11_ESM.docx]

**Additional file 11.** Univariate and multivariable meta-regression analyses to identify sources of heterogeneity in studies reporting on pre-DM prevalence in women of childbearing age by the different measured characteristics

|  | **No. of studies** | **Univariate analyses** | | **Multivariable analyses** | |
| --- | --- | --- | --- | --- | --- |
|  |  | OR (95% CI) | *P*–value^†^ | aOR (95% CI) | *P*–value^‡^ |
| **Country** |  |  |  |  |  |
| Iran | 13 | 0.47 (0.21–1.10) | 0.078 | 0.30 (0.11–0.79) | 0.017 |
| Iraq | 3 | 5.24 (1.45–18.94) | 0.013 | 2.20 (0.52–10.82) | 0.251 |
| Jordan | 1 | 2.40 (0.31–18.50) | 0.394 | 2.63 (0.18–37.4) | 0.464 |
| Kuwait | 4 | 2.20 (0.69–6.99) | 0.174 | 2.69 (0.61–11.83) | 0.182 |
| Morocco | 2 | 0.21 (0.03–1.61) | 0.129 | 0.12 (0.01–0.91) | 0.041 |
| Oman | 3 | 0.67 (0.18–2.42) | 0.535 | 1.05 (0.18–6.13) | 0.950 |
| Qatar | 3 | 0.35 (0.45–2.71) | 0.307 | 0.55 (0.05–5.65) | 0.606 |
| Saudi Arabia^§^ | 11 | 1.00 |  | 1.00 |  |
| United Arab Emirates | 11 | 2.20 (0.93–5.13) | 0.071 | 1.91 (0.38–9.78) | 0.422 |
| Yemen | 1 | 0.56 (0.07–4.34) | 0.570 | 0.88 (0.09–9.03) | 0.912 |
| **Population** |  |  |  |  |  |
| General population^5^ | 45 | 1.00 |  | 1.00 |  |
| Pregnant | 4 | 0.69 (0.19–2.42) | 0.558 | – |  |
| Non-pregnant with a history of GDM | 3 | 0.50 (0.12–2.08) | 0.332 | – |  |
| **Study period**^7^ |  |  |  |  |  |
| 2000–2009 | 35 | 1.00 |  | 1.00 |  |
| 2010–2018 | 12 | 1.72 (0.77–3.85) | 0.180 | 1.42 (0.44–4.65) | 0.544 |
| Overlapping^8^ | 5 | 2.90 (0.96–8.77) | 0.059 | 1.40 (0.28–6.94) | 0.672 |
| **Ascertainment**^9^ |  |  |  |  |  |
| Medical records/anti-DM medications/self-reported | 7 | 1.00 |  | 1.00 |  |
| WHO guidelines | 19 | 1.92 (0.64–5.78) | 0.238 | 1.30 (0.16–10.25) | 0.804 |
| ADA guidelines | 23 | 2.56 (0.88–7.49) | 0.085 | 3.51 (0.79–15.57) | 0.094 |
| Carpenter and Coustan | 3 | 0.84 (0.16–4.34) | 0.834 | 3.27 (0.49–21.57) | 0.210 |
| **Sample size** |  |  |  |  |  |
| <100 | 10 | 1.00 |  | 1.00 |  |
| ≥100 | 42 | 1.06 (0.40–2.83) | 0.896 | – |  |

^†^ Statistically significant at p-values <0.1

^‡^ Statistically significant at p-values <0.05

^§^ Reference country

^¶^ Year range in square brackets does not cover every single year within that range.

OR: odds ratio; aOR: adjusted odds ratio; CI, confidence interval calculated using the “exact” method.

CI, confidence interval calculated using the “exact” method.

Pre-DM: pre diabetes mellitus; GDM: gestational diabetes; WHO: World Health Organization; ADA: American Diabetes Association; IDF: International Diabetes Federation
