# Supplementary material for: Type 2 diabetes and pre-diabetes mellitus: a systematic review and meta-analysis of prevalence studies in women of childbearing age in the Middle East and North Africa, 2000–2018
Source: Syst Rev. 2019 Nov 8;8:268. doi: 10.1186/s13643-019-1187-1 (PMC6839168; doi:10.1186/s13643-019-1187-1)
Supplement: Supplementary file 12 — Additional file 12. Quality assessment of the 48 research reports included in the analysis. [file 13643_2019_1187_MOESM12_ESM.docx]

**Additional file 12**. Quality assessment of the 48 research reports included in the analysis

| **Author** | **Q 1** | **Q 2** | **Q 3** | **Q 4** | **Q 5** | **Q 6** | **Q 7** | **Q 8** | **Q 9** | **Overall score* / 9** |
| --- | --- | --- | --- | --- | --- | --- | --- | --- | --- | --- |
| Eldesoky AE. et al., 2013 [1] | 1 | 1 | 1 | 1 | 0 | 1 | 1 | 3 | 0 | 6 |
| Gunaid AA. and Assabri AM., 2008 [2] | 1 | 1 | 1 | 1 | 1 | 1 | 1 | 1 | 0 | 8 |
| Azimi–Nezhad M. et al., 2008 [3] | 1 | 1 | 2 | 1 | 0 | 1 | 1 | 1 | 1 | 6 |
| Bener A. et al., 2009 [4] | 1 | 1 | 1 | 1 | 1 | 1 | 1 | 1 | 1 | 9 |
| Baynouna LM. et al., 2008 [5] | 1 | 1 | 0 | 1 | 0 | 1 | 1 | 1 | 1 | 8 |
| Azimi–Nezhad M. et al., 2009 [6] | 1 | 1 | 4 | 1 | 2 | 1 | 3 | 1 | 1 | 6 |
| Hossein–nezhad A. et al., 2009 [7] | 1 | 1 | 2 | 1 | 0 | 1 | 1 | 1 | 1 | 6 |
| Chodick G. et al., 2003 [8] | 1 | 1 | 1 | 1 | 0 | 1 | 1 | 3 | 1 | 7 |
| Karim A. et al., 2000 [9] | 1 | 1 | 4 | 1 | 0 | 4 | 1 | 1 | 1 | 6 |
| Alqurashi KA. et al., 2011 [10] | 1 | 1 | 1 | 1 | 0 | 1 | 0 | 1 | 1 | 7 |
| Al–Baghli NA. et al., 2010 [11] | 1 | 1 | 0 | 1 | 2 | 1 | 1 | 3 | 1 | 6 |
| Salima T. et al., 2011 [12] | 1 | 1 | 2 | 1 | 0 | 1 | 1 | 3 | 1 | 6 |
| Abu–Zaiton A and Al–Fawwaz A. 2013 [13] | 1 | 1 | 2 | 1 | 0 | 1 | 1 | 1 | 0 | 6 |
| Mansour AA. et al., 2014 [14] | 1 | 1 | 4 | 1 | 0 | 1 | 1 | 3 | 1 | 6 |
| Hadaegh F. et al., 2008 [15] | 1 | 1 | 1 | 1 | 1 | 1 | 1 | 3 | 1 | 8 |
| Saadi H. et al., 2007 [16] | 1 | 1 | 0 | 1 | 1 | 2 | 1 | 1 | 1 | 7 |
| Wahabi HA. et al., 2012 [17] | 1 | 1 | 2 | 1 | 2 | 1 | 1 | 3 | 1 | 6 |
| Gowri V. et al., 2011 [18] | 1 | 1 | 3 | 1 | 0 | 1 | 3 | 1 | 1 | 6 |
| Al–Lawati JA. et al., 2002 [19] | 1 | 1 | 1 | 1 | 0 | 1 | 1 | 1 | 1 | 8 |
| Ebrahimi H. et al., 2016 [20] | 1 | 1 | 1 | 1 | 0 | 1 | 1 | 1 | 1 | 8 |
| Al Dhaheri A. et al., 2016 [21] | 1 | 1 | 1 | 1 | 1 | 1 | 1 | 1 | 1 | 9 |
| Bahijri S. et al., 2016 [22] | 1 | 1 | 1 | 1 | 1 | 1 | 1 | 1 | 1 | 9 |
| Ben Romdhane H. et al., 2014 [23] | 1 | 1 | 1 | 1 | 1 | 1 | 3 | 1 | 1 | 8 |
| Amin T. et al., 2014 [24] | 1 | 1 | 0 | 1 | 0 | 1 | 1 | 0 | 1 | 6 |
| Ahmed F. et al., 2013 [25] | 1 | 1 | 1 | 1 | 0 | 1 | 1 | 0 | 1 | 7 |
| Hajat C. et al., 2012 [26] | 1 | 1 | 1 | 1 | 0 | 1 | 1 | 3 | 1 | 7 |
| Saeed A., et al 2017 [27] | 1 | 1 | 2 | 1 | 2 | 1 | 1 | 1 | 1 | 7 |
| Al–Daghri N. et al., 2011 [28] | 1 | 1 | 1 | 1 | 0 | 1 | 1 | 1 | 1 | 8 |
| Alattar A. et al., 2012 [29] | 1 | 1 | 4 | 1 | 0 | 1 | 1 | 1 | 1 | 7 |
| Mansour A. et al., 2008 [30] | 1 | 1 | 2 | 1 | 0 | 1 | 1 | 1 | 0 | 6 |
| Shah S. et al., 2017 [31] | 1 | 1 | 1 | 1 | 1 | 1 | 1 | 1 | 1 | 9 |
| Al Serehi A. et al., 2015 [32] | 1 | 1 | 4 | 1 | 0 | 1 | 1 | 3 | 1 | 6 |
| Agarwal MM. et al., 2004 [33] | 1 | 1 | 4 | 1 | 0 | 1 | 1 | 3 | 1 | 6 |
| Al–Rubeaan K. et al., 2015 [34] | 1 | 1 | 2 | 1 | 0 | 1 | 1 | 1 | 1 | 7 |
| Valizadeh MH. et al., 2015 [35] | 1 | 1 | 1 | 1 | 0 | 1 | 1 | 3 | 1 | 7 |
| Al-Qahtani D. et al., 2006 [36] | 1 | 1 | 1 | 1 | 0 | 1 | 1 | 1 | 1 | 8 |
| Rguibi M. and Belahsen R., 2005 [37] | 1 | 1 | 2 | 1 | 0 | 1 | 1 | 1 | 1 | 7 |
| Shaaban LA. et al., 2006 [38] | 1 | 1 | 1 | 1 | 0 | 1 | 3 | 1 | 1 | 7 |
| Agarwal MM. et al., 2015 [39] | 1 | 1 | 1 | 1 | 0 | 1 | 1 | 1 | 1 | 8 |
| Tohme R. et al., 2005 [40] | 1 | 1 | 1 | 1 | 1 | 1 | 3 | 1 | 1 | 8 |
| Keshavarz M. et al., 2005 [41] | 1 | 1 | 1 | 1 | 0 | 1 | 1 | 1 | 0 | 7 |
| Malik M. et al., 2005 [42] | 1 | 1 | 1 | 1 | 0 | 1 | 1 | 1 | 1 | 8 |
| Al-Nazhan AS. et al., 2017 [43] | 1 | 1 | 1 | 1 | 0 | 1 | 3 | 1 | 1 | 7 |
| Al–Rubeaan K. et al., 2014 [44] | 1 | 1 | 1 | 1 | 0 | 1 | 1 | 1 | 1 | 8 |
| Habib AF. et al., 2002 [45] | 1 | 1 | 1 | 1 | 0 | 1 | 1 | 1 | 1 | 8 |
| Diejomaoh M. et al., 2007 [46] | 1 | 1 | 1 | 1 | 0 | 1 | 1 | 1 | 0 | 7 |
| Saeed A., 2012 [47] | 1 | 1 | 1 | 1 | 1 | 1 | 3 | 1 | 1 | 8 |
| Sulaiman N. et al., 2018 [48] | 1 | 1 | 1 | 1 | 2 | 1 | 1 | 1 | 1 | 8 |
| **% of research reports meeting the item** | **100%** | **100%** | **58.3%** | **100%** | **20.8%** | **95.8%** | **83.3%** | **72.9%** | **87.5%** | **Average: 7.2**** |

Q1: Was the research question or objective in this paper clearly stated? 1: Low Risk of bias (ROB), 2: High ROB, 3: Unclear ROB

Q2. Was the study population clearly specified and defined? 1: ROB, 2: High ROB, 3: Unclear ROB

Q 3. Was the participation rate of eligible persons at least 50%? 1: ROB, 2: High ROB, 3: Unclear ROB

Q4. Were all the subjects selected or recruited from the same or similar populations (including the same time period)? Were inclusion and exclusion criteria for being in the study prespecified and applied uniformly to all participants? 1: Low ROB, 2: High ROB, 3: Unclear ROB

Q5. Was a sample size justification, power description, or variance and effect estimates provided? 1: ROB, 2: High ROB, 3: Unclear ROB

Q6. Were the outcome measures (dependent variables) clearly defined, valid, reliable, and implemented consistently across all study participants? 1: Low ROB, 2: High ROB, 3: Unclear ROB

Q7: DM ascertainment (1: Biological assay; 2:self-reported; 3: unclear)

Q8: Sampling methodology (1: Probability-based; 2: non-probability based; 3: Unclear)

Q9: Precision (1: tested sample size ≥100; 2: tested sample size<100)

* How many items out of the nine measured items were met in each specific study?

** Average number of items met across the 48 studies**.**
